# Tetra­phenyl­phospho­nium iodide–1,3,5-tri­fluoro-2,4,6-tri­iodo­benzene–methanol (3/4/1)

**DOI:** 10.1107/S1600536813012397

**Published:** 2013-05-11

**Authors:** Gabriella Cavallo, Pierangelo Metrangolo, Tullio Pilati, Giuseppe Resnati, Giancarlo Terraneo

**Affiliations:** a NFMLab, Department of Chemistry, Materials and Chemical Engineering, "Giulio Natta", Politecnico di Milano, Via Mancinelli, 7, I-20131 Milano, Italy

## Abstract

The crystallization of a 1:1 molar solution of 1,3,5-tri­fluoro-2,4,6-di­iodo­benzene (TFTIB) and tetra­phenyl­phosponium iodide (TPPI) from methanol produced tetra­gonal needles of pure TPPI and tabular pseudo-hexa­gonal truncated bipyramids of the title compound, 3C_24_H_20_P^+^·3I^−^·4C_6_F_3_I_3_·CH_4_O or (TPPI)_3_(TFTIB)_4_·MeOH. The asymmetric unit is composed of six TPPI mol­ecules, eight TFTIB mol­ecules and two methanol mol­ecules, overall 16 constituents. The formation of the architecture is essentially guided by a number of C—I⋯I^−^ halogen bonds (XB), whose lengths are in the range 3.276 (1)–3.625 (1) Å. Layers of supra­molecular polyanions are formed parallel to (10-1) wherein iodide anions function as penta-, tetra- or bidentate XB acceptors. The structure is not far from being *P*2_1_/*n*, but the centrosymmetry is lost due to a different conformation of a single couple of cations and the small asymmetry in the formed supra­molecular anion. One methanol mol­ecule is hydrogen bonded to an iodide anion, while the second is linked to the first one *via* an O—H⋯O contact. This second methanol mol­ecule is more loosely pinned in its position than the first and presents very high anisotropic displacement parameters and a seeming shortening of the C—O bond length. The crystal studied was refined as a perfect inversion twin.

## Related literature

For the structure of pure TPPI, see: Schweizer *et al.* (1989 ▶), for the structure of pure TFTIB, see: Nath *et al.* (2008 ▶); Reddy *et al.* (2006 ▶) and for the structure of TPPI/TFTIB·CHCl_3_, see: Metrangolo *et al.* (2008 ▶, 2009 ▶). For the use of TFTIB in crystal engineering based on halogen bonding, see: Lucassen *et al.* (2007 ▶).
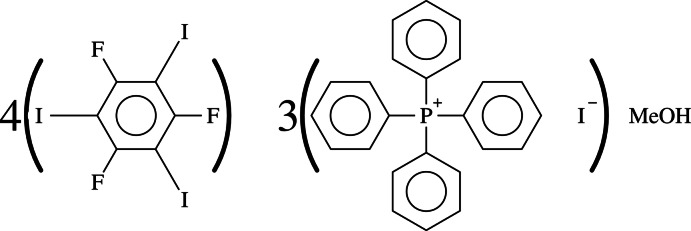


## Experimental

### 

#### Crystal data

3C_24_H_20_P^+^·3I^−^·4C_6_F_3_I_3_·CH_4_O
*M* *_r_* = 3469.89
Monoclinic, 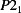

*a* = 17.233 (3) Å
*b* = 22.001 (4) Å
*c* = 28.260 (5) Å
β = 92.49 (2)°
*V* = 10704 (3) Å^3^
*Z* = 4
Mo *K*α radiation
μ = 4.45 mm^−1^
*T* = 90 K
0.34 × 0.20 × 0.12 mm

#### Data collection

Bruker SMART APEX diffractometer
Absorption correction: multi-scan (*SADABS*; Bruker, 1998) ▶ *T*_min_ = 0.614, *T*_max_ = 1.000
105822 measured reflections
28547 independent reflections
26385 reflections with *I* > 2σ(*I*)
*R*_int_ = 0.047

#### Refinement

*R*[*F* ^2^ > 2σ(*F* ^2^)] = 0.040
*wR*(*F* ^2^) = 0.081
*S* = 1.09
28547 reflections
2304 parameters
13 restraints
H-atom parameters constrained
Δρ_max_ = 2.46 e Å^−3^
Δρ_min_ = −0.89 e Å^−3^

### 

Data collection: *APEX2* (Bruker, 1998 ▶); cell refinement: *SAINT* (Bruker, 1998 ▶); data reduction: *SAINT*; program(s) used to solve structure: *SIR2002* (Burla *et al.*, 2003 ▶); program(s) used to refine structure: *SHELXL2012* (Sheldrick, 2008) ▶; molecular graphics: *ORTEP-3 for Windows* (Farrugia, 2012 ▶) and *Mercury* (Macrae *et al.*, 2006 ▶); software used to prepare material for publication: *SHELXL2012*.

## Supplementary Material

Crystal structure: contains datablock(s) global, I. DOI: 10.1107/S1600536813012397/im2426sup1.cif

Structure factors: contains datablock(s) I. DOI: 10.1107/S1600536813012397/im2426Isup2.hkl

Additional supplementary materials:  crystallographic information; 3D view; checkCIF report

## Figures and Tables

**Table 1:** Hydrogen-bond geometry (Å, °)

*D*—H⋯*A*	*D*—H	H⋯*A*	*D*⋯*A*	*D*—H⋯*A*
C20*I*—H20*I*⋯I3^i^	0.95	3.16	3.866 (9)	133
C20*L*—H20*L*⋯I6	0.95	3.09	3.821 (9)	135
C14*M*—H14*M*⋯I3^ii^	0.95	3.14	3.788 (9)	127
C8*L*—H8*L*⋯F3*F* ^i^	0.95	2.59	3.351 (10)	138
C20*L*—H20*L*⋯I6	0.95	3.09	3.821 (9)	135
C18*M*—H18*M*⋯F2*C* ^iii^	0.95	2.56	3.257 (11)	130
C18*L*—H18*L*⋯O2*S*	0.95	2.61	3.25 (2)	125
O1*S*—H1*S*⋯I1	0.85	2.71	3.561 (15)	177
O2*S*—H2*S*⋯O1*S*	0.85	1.83	2.68 (3)	176

Symmetry codes: (i) 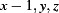; (ii) 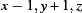; (iii) 
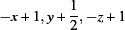
.

**Table 2:** Short C—I⋯I^−^ contacts and XBs (Å, °) The table is organized to evidence the difference between the two pseudo-centrosymmetric units: similar contacts are on the same line and are also reported even if they are too long to be considered as XBs.

C—*X*⋯*Y*	*X*⋯*Y*	C—*X*⋯*Y*	C—*X*⋯*Y*	*X*⋯*Y*	C—*X*⋯*Y*
C1*A*—I1*A*⋯I1	3.5318 (10)	175.26 (3)	C1*E*—I1*E*⋯I4	3.5567 (10)	177.55 (3)
C3*E*—I2*E*⋯I1	3.6860 (10)	169.40 (3)	C3*A*—I2*A*⋯I4	3.5578 (11)	173.31 (3)
C1*B*—I1*B*⋯I1	3.4466 (9)	169.04 (3)	C1*F*—I1*F*⋯I4	3.4131 (10)	168.78 (3)
C3*G*—I2*G*⋯I1	3.4582 (10)	169.32 (2)	C3*C*—I2*C*⋯I4	3.4102 (10)	172.40 (3)
C5*A*—I3*A*⋯I2	3.4725 (10)	167.88 (3)	C5*E*—I3*E*⋯I5	3.4221 (10)	174.57 (3)
C3*D*—I2*D*⋯I2	3.4963 (10)	174.57 (3)	C3*H*—I2*H*⋯I5	3.5754 (10)	168.90 (3)
C5*B* ^i^—I3*B* ^i^⋯I2	3.5678 (10)	171.87 (3)	C5*F* ^ii^—I3*F* ^ii^⋯I5	3.5696 (10)	160.64 (3)
C5*C* ^i^—I3*C* ^i^⋯I2	3.5029 (9)	164.46 (3)	C5*G* ^ii^—I3*G* ^ii^⋯I5	3.6253 (10)	157.18 (3)
C5*D* ^i^—I3*D* ^i^⋯I2	3.6906 (10)	176.88 (3)	C5*H* ^ii^—I3*H* ^ii^⋯I5	4.0145 (11)	166.52 (3)
C1*C*—I1*C*⋯I3	3.2760 (9)	179.47 (3)	C1*H* ^ii^—I1*H* ^ii^⋯I3	3.3977 (10)	178.17 (3)
C1*G*—I1*G*⋯I6	3.2889 (9)	172.31 (3)	C1*D* ^i^—I1*D* ^i^⋯I6	3.4609 (10)	172.84 (3)

Symmetry codes: (i) 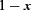, 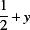, 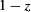; (ii) 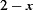, 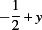, 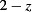.

**Fig. 1.**
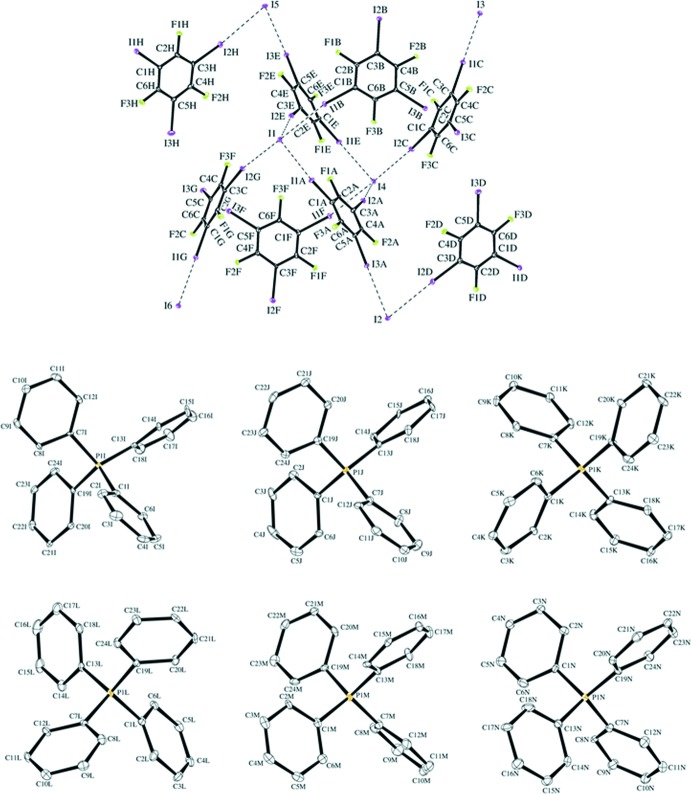
The various components of the title complex with thermal ellipsoids at the 50% probability level. The six TPP+ cations are plotted in different projections to avoid confusing overpositions. The 8(C6F3I3).6I- unit is projected on a single plane. Methanol molecules are omitted.

**Fig. 2.**
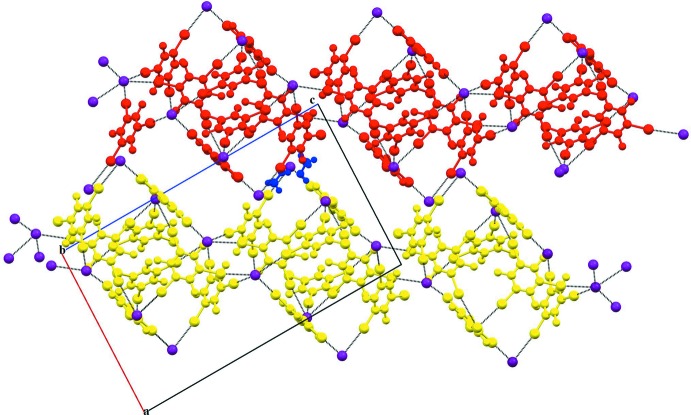
Ball and stick representation (Mercury 3.1) of two layers of supramolecular polyanions: Iodide anions are in violet, all other atoms are in red in one layer and in yellow in the other; two methanol molecules are in blue; XBs are dotted black lines; cations are omitted for sake of simplicity.

## supplementary crystallographic information

### Comment

Anion coordination chemistry has been systematically investigated during the years since the full understanding and exploitation could impact in different research fields such biosciences, advanced materials and catalysis. Hydrogen bonding has been extensively used in anion coordination. More recently also halogen bonding has shown its efficiency and reliability in the anion coordination field and now could be considered the first-choice tool for driving anions recognition processes (Metrangolo *et al.* 2009). 1,3,5-trifluoro,2,4,6-diiodobenzene (TFTIB) has been used as tridentate halogen bonding donor unit *versus* the tetraphenylphosponium iodide (TPPI, Schweizer *et al.* 1989) where the iodide atom functions as halogen bonding acceptor. Halide anions as iodide are spherical anions and can accept multiple halogen bonding donors. Thanks to the threefold symmetry module TFTIB and the possible three-coordinate profile of iodide a (6,3) networks could be formed if the empty space inside the hexagonal framework is opportunely filled by a counterion of the right size. Tetraphenylphosponium is larger than the ammonium cation (Metrangolo *et al.*, 2008) and therefore prevents the translation of the starting modules with trigonal symmetry into the formation of a (6,3) networks supramolecular network. The asymmetric unit is composed by six TPPI molecules, four TFTIB molecules and two methanol molecules, overall 16 costituents. The formation of the architecture is essentially guided by a number of C—I···I^-^ halogen bonds (XB) whose distances vary in the range 3.276 (1) - 3.625 (1) Å. Layers of supramolecular polyanions are formed wherein iodide anions function as penta-, tetra- or bi-dentate XB acceptors. The structure is not far from being *P* 2_1_/*n*, but the centrosymmetry is lost due to a different conformation of a single couple of cations and the small asymmetry in the formed supramolecular anion.

### Experimental

The complex was crystallized from a methanol solution. The crystal used for data collection was cut from a very large pseudo-hexagonal plate. The batch contained also long tetragonal needles of pure tetraphenylphosphonium iodide. The temperature was controlled by a low temperature OXFORD device.

### Refinement

Refined as a 2-component perfect inversion twin. The refinement without inversion twin gave ambiguous results: the direct and the inverted model ended with Flack parameters 0.131 (14) and 0.126 (14), respectively; the final results of this models were nearly indistinguishable in terms of *R*, *wR*, Δρ. We then adopted the perfect inversion twin hypothesis, also because the final weighting scheme gave a smaller second term: 2.7391 *versus* 4.4365 and 4.683, respectively, for the direct and inverted, not twinned, models. The packing analysis with Mercury shows two holes. Looking at the final 400 residues, only two peaks, with low ranking, namely Q239 and Q323 were compatible with the largest holes and none with the smaller one; an attempt to refine these peaks as a methanol molecule, using restraints on the C—O distance and on isotropic displacement parameters, converged to a value of about 0.1 for the population factor, that is something corresponding to a bit more than one electron. As a consequence, we decided to ignore these peaks. Residual peaks Q1 and Q2 are located near to the iodine atoms on TFTIB, Q1 is closed to I3H and Q2 is closed to I3D. This is due to imperfect absorption correction correlated to the high resolution data. We have carefully examined a second question: a possible higher symmetry. We have designed all the molecules with a suffix, from a to A to H for the TFTIB ones, from I to N for the TPP^+^ cations. The mass centres of all the couples A/E, B/F, C/G, D/H, I/*L*, J/*M*, K/N, I1/I4, I2/I5, I3/I6 is at about 0.50(or 1.50), 1.00 1.50 and this is compatible with a center of symmetry located at 1/4,0,0.75. Only for the couples K/N and I2/I5 this is not exactly true: for K/N because the inversion of N do not give exactly *L*, but two of the four phenyl groups are largely rotated; for I1/I3 because their mass center is 1.51, 1.03, 1.50, largely different from a possible center of symmetry of the whole structure. At the same time, while the second methanol molecule (suffix 2S) is placed about on the possible inversion center, the first one (1*S*) does not have a correspondence with others. Moreover, the possible centrosymmetry is invalidated by the intensities data. In fact, assuming the space group *P*2_1_/*n*, there are 423 systematic absences violations over 680 with k=0 and h+l odd, and the situation is worse if *P*2_1_/*a* or *P*2_1_/*c* space groups are considered. Notwithstanding, we tried to refine the structure as *P*2_1_/*n*, after translation of 1/4, 0, 3/4, elimination of the second member of any couple, excluding K/N for which was assumed the disorder with population factor 0.5/0.5, as made for the 2S methanol molecule, while for 1S one we refined the population factor. The results, for the refinement in the space group *P*2_1_/*n*, where absurd: *R*_1_ near 1/5, w*R*_2_ more than the double, the second terms of weighting scheme suggested by *SHELXL* around 3000, very large and positive (Io—Ic) residues, non-positive definite ADPs. As a consequence, we think that the *P*2_1_ space group is, with no doubt, the only possible choice. The center of pseudo-symmetry is situated at 3/4, 1/2, 0.75. Hydrogen atoms were positioned geometrically and refined using a riding model, with C—H = 0.95–0.99 Å and with *U*_iso_(H) = 1.2 (1.5 for hydroxyl group) times *U*_eq_(C).

### Crystal data

**Table:**

3C_24_H_20_P^+^·3I^−^·4C_6_F_3_I_3_·CH_4_O	*F*(000) = 6408
*M**_r_* = 3469.89	*D*_x_ = 2.153 Mg m^−^^3^
Monoclinic, *P*2_1_	Mo *K*α radiation, λ = 0.71073 Å
*a* = 17.233 (3) Å	Cell parameters from 23933 reflections
*b* = 22.001 (4) Å	θ = 2.3–28.2°
*c* = 28.260 (5) Å	µ = 4.45 mm^−^^1^
β = 92.49 (2)°	*T* = 90 K
*V* = 10704 (3) Å^3^	Irregular prism, colourless
*Z* = 4	0.34 × 0.20 × 0.12 mm

### Data collection

**Table:**

Bruker SMART APEX diffractometer	26385 reflections with *I* > 2σ(*I*)
Radiation source: sealed tube	*R*_int_ = 0.047
phi and ω scans	θ_max_ = 29.5°, θ_min_ = 1.5°
Absorption correction: multi-scan (*SADABS*; Bruker, 1998)	*h* = −23→23
*T*_min_ = 0.614, *T*_max_ = 1.000	*k* = −30→30
105822 measured reflections	*l* = −38→38
28547 independent reflections	

### Refinement

**Table:**

Refinement on *F*^2^	Primary atom site location: structure-invariant direct methods
Least-squares matrix: full	Secondary atom site location: difference Fourier map
*R*[*F*^2^ > 2σ(*F*^2^)] = 0.040	Hydrogen site location: inferred from neighbouring sites
*wR*(*F*^2^) = 0.081	H-atom parameters constrained
*S* = 1.09	*w* = 1/[σ^2^(*F*_o_^2^) + (0.0397*P*)^2^ + 2.7944*P*] where *P* = (*F*_o_^2^ + 2*F*_c_^2^)/3
28547 reflections	(Δ/σ)_max_ = 0.002
2304 parameters	Δρ_max_ = 2.46 e Å^−^^3^
13 restraints	Δρ_min_ = −0.89 e Å^−^^3^

### Special details

**Table:**

Geometry. All e.s.d.'s are estimated using the full covariance matrix. The cell e.s.d.'s are taken into account individually in the estimation of e.s.d.'s in distances, angles and torsion angles; correlations between e.s.d.'s in cell parameters are used only when they are defined by crystal symmetry.

### Fractional atomic coordinates and isotropic or equivalent isotropic displacement parameters (Å^2^)

**Table:**

	*x*	*y*	*z*	*U*_iso_*/*U*_eq_	
I1	0.48788 (4)	0.42157 (3)	0.87134 (2)	0.02089 (12)	
I2	0.35374 (3)	0.60861 (3)	0.45407 (2)	0.02348 (13)	
I3	1.27368 (3)	0.08604 (3)	0.68027 (2)	0.02004 (12)	
I4	0.99320 (4)	0.58570 (3)	0.63416 (2)	0.02230 (12)	
I5	1.15821 (3)	0.42256 (3)	1.04672 (2)	0.02239 (12)	
I6	0.22490 (3)	0.91480 (3)	0.81920 (2)	0.01906 (11)	
I1A	0.53202 (4)	0.48501 (3)	0.76090 (2)	0.02227 (12)	
I2A	0.78941 (3)	0.56014 (3)	0.63496 (2)	0.02262 (12)	
I3A	0.45612 (3)	0.56198 (3)	0.55623 (2)	0.02090 (12)	
F1A	0.6947 (3)	0.5224 (2)	0.72370 (17)	0.0232 (11)	
F2A	0.6384 (3)	0.5722 (3)	0.56453 (17)	0.0258 (12)	
F3A	0.4378 (3)	0.5137 (3)	0.66250 (18)	0.0261 (12)	
C1A	0.5646 (5)	0.5166 (4)	0.6948 (3)	0.0220 (19)	
C2A	0.6435 (5)	0.5285 (4)	0.6870 (3)	0.0182 (18)	
C3A	0.6700 (5)	0.5471 (4)	0.6440 (3)	0.0203 (18)	
C4A	0.6149 (5)	0.5538 (4)	0.6071 (3)	0.0190 (18)	
C5A	0.5367 (5)	0.5454 (4)	0.6126 (3)	0.0206 (19)	
C6A	0.5143 (5)	0.5261 (4)	0.6564 (3)	0.0211 (19)	
I1B	0.56506 (3)	0.28204 (3)	0.84399 (2)	0.02083 (12)	
I2B	0.74521 (3)	0.04470 (3)	0.86393 (2)	0.02143 (12)	
I3B	0.63264 (3)	0.13315 (3)	0.67009 (2)	0.01979 (12)	
F1B	0.6519 (3)	0.1648 (2)	0.88909 (17)	0.0239 (12)	
F2B	0.7098 (3)	0.0536 (2)	0.75318 (17)	0.0232 (11)	
F3B	0.5750 (3)	0.2382 (2)	0.73825 (18)	0.0249 (12)	
C1B	0.6145 (6)	0.2043 (4)	0.8144 (3)	0.0213 (19)	
C2B	0.6496 (5)	0.1595 (4)	0.8418 (3)	0.0180 (18)	
C3B	0.6843 (4)	0.1086 (4)	0.8220 (3)	0.0168 (17)	
C4B	0.6786 (5)	0.1034 (4)	0.7734 (3)	0.0179 (18)	
C5B	0.6418 (5)	0.1457 (4)	0.7440 (3)	0.0139 (16)	
C6B	0.6111 (5)	0.1954 (4)	0.7659 (3)	0.0186 (18)	
I1C	1.13008 (3)	0.17623 (3)	0.64724 (2)	0.01700 (11)	
I2C	0.99571 (3)	0.43226 (3)	0.61722 (2)	0.02144 (12)	
I3C	0.79691 (3)	0.21021 (3)	0.57610 (2)	0.02054 (12)	
F1C	1.1147 (3)	0.3228 (2)	0.6401 (2)	0.0271 (12)	
F2C	0.8552 (3)	0.3475 (2)	0.58535 (18)	0.0221 (11)	
F3C	0.9579 (3)	0.1522 (2)	0.60831 (19)	0.0238 (12)	
C1C	1.0374 (5)	0.2353 (4)	0.6253 (3)	0.0171 (17)	
C2C	1.0454 (5)	0.2989 (4)	0.6259 (3)	0.0153 (17)	
C3C	0.9851 (5)	0.3378 (4)	0.6129 (3)	0.0210 (19)	
C4C	0.9168 (5)	0.3118 (4)	0.5980 (3)	0.0208 (19)	
C5C	0.9036 (5)	0.2490 (4)	0.5971 (3)	0.0151 (16)	
C6C	0.9663 (5)	0.2121 (4)	0.6099 (3)	0.0202 (18)	
I1D	0.70112 (3)	0.39095 (3)	0.29119 (2)	0.02127 (12)	
I2D	0.46797 (3)	0.48346 (3)	0.42597 (2)	0.02289 (12)	
I3D	0.62780 (4)	0.24274 (3)	0.46782 (2)	0.03187 (15)	
F1D	0.5743 (3)	0.4689 (2)	0.33943 (18)	0.0246 (12)	
F2D	0.5162 (3)	0.3557 (3)	0.47358 (18)	0.0313 (13)	
F3D	0.7077 (3)	0.2874 (3)	0.3733 (2)	0.0319 (13)	
C1D	0.6430 (6)	0.3783 (4)	0.3535 (3)	0.023 (2)	
C2D	0.5865 (5)	0.4198 (4)	0.3668 (3)	0.0183 (17)	
C3D	0.5445 (5)	0.4137 (4)	0.4063 (3)	0.0223 (19)	
C4D	0.5575 (6)	0.3628 (5)	0.4349 (3)	0.024 (2)	
C5D	0.6108 (6)	0.3194 (4)	0.4241 (3)	0.025 (2)	
C6D	0.6530 (5)	0.3285 (4)	0.3842 (3)	0.0216 (19)	
I1E	0.94880 (3)	0.53018 (3)	0.74804 (2)	0.02023 (12)	
I2E	0.70087 (3)	0.43824 (3)	0.87455 (2)	0.02251 (12)	
I3E	1.03676 (3)	0.44957 (3)	0.94998 (2)	0.01902 (12)	
F1E	0.7889 (3)	0.4850 (2)	0.78615 (17)	0.0225 (11)	
F2E	0.8554 (3)	0.4246 (3)	0.94140 (17)	0.0258 (12)	
F3E	1.0459 (3)	0.5047 (2)	0.84530 (17)	0.0212 (11)	
C1E	0.9178 (5)	0.4964 (4)	0.8141 (3)	0.0208 (19)	
C2E	0.8435 (5)	0.4781 (4)	0.8222 (3)	0.0173 (17)	
C3E	0.8193 (5)	0.4568 (4)	0.8646 (3)	0.0194 (18)	
C4E	0.8754 (5)	0.4481 (4)	0.8995 (3)	0.0216 (19)	
C5E	0.9531 (5)	0.4632 (4)	0.8941 (3)	0.0210 (19)	
C6E	0.9722 (5)	0.4882 (4)	0.8515 (3)	0.0179 (17)	
I1F	0.92817 (3)	0.72748 (3)	0.66130 (2)	0.02239 (12)	
I2F	0.74139 (4)	0.96267 (3)	0.64622 (2)	0.02765 (14)	
I3F	0.88425 (4)	0.88532 (3)	0.83422 (2)	0.02367 (13)	
F1F	0.8317 (3)	0.8415 (3)	0.61781 (17)	0.0276 (13)	
F2F	0.7977 (3)	0.9593 (2)	0.75376 (17)	0.0224 (11)	
F3F	0.9392 (3)	0.7780 (2)	0.76541 (18)	0.0249 (12)	
C1F	0.8849 (5)	0.8085 (4)	0.6916 (3)	0.0187 (18)	
C2F	0.8428 (5)	0.8504 (4)	0.6649 (3)	0.0191 (18)	
C3F	0.8112 (5)	0.9015 (4)	0.6852 (3)	0.0158 (17)	
C4F	0.8269 (5)	0.9101 (4)	0.7332 (3)	0.0206 (19)	
C5F	0.8692 (6)	0.8698 (4)	0.7614 (3)	0.0212 (19)	
C6F	0.8972 (5)	0.8186 (4)	0.7394 (3)	0.0191 (18)	
I1G	0.37461 (3)	0.82873 (2)	0.85027 (2)	0.01662 (11)	
I2G	0.50870 (3)	0.57598 (2)	0.89093 (2)	0.01950 (12)	
I3G	0.71177 (3)	0.80152 (3)	0.91375 (2)	0.02290 (12)	
F1G	0.3906 (3)	0.6839 (2)	0.86649 (19)	0.0234 (11)	
F2G	0.6546 (3)	0.6628 (2)	0.91318 (18)	0.0228 (11)	
F3G	0.5507 (3)	0.8559 (2)	0.87860 (18)	0.0208 (11)	
C1G	0.4685 (5)	0.7711 (4)	0.8710 (3)	0.0169 (17)	
C2G	0.4618 (5)	0.7088 (5)	0.8758 (3)	0.024 (2)	
C3G	0.5223 (5)	0.6711 (4)	0.8891 (3)	0.0156 (17)	
C4G	0.5937 (5)	0.6979 (4)	0.8992 (3)	0.0182 (18)	
C5G	0.6062 (5)	0.7599 (4)	0.8954 (3)	0.0178 (18)	
C6G	0.5414 (5)	0.7948 (4)	0.8818 (3)	0.0172 (17)	
I1H	0.79723 (3)	0.61341 (3)	1.21145 (2)	0.02091 (12)	
I2H	1.03764 (3)	0.54967 (3)	1.07214 (2)	0.02596 (13)	
I3H	0.84358 (5)	0.77698 (4)	1.03897 (3)	0.04102 (18)	
F1H	0.9330 (3)	0.5503 (2)	1.15952 (18)	0.0238 (11)	
F2H	0.9701 (3)	0.6751 (3)	1.02808 (19)	0.0356 (14)	
F3H	0.7745 (3)	0.7220 (3)	1.1333 (2)	0.0332 (14)	
C1H	0.8539 (5)	0.6365 (4)	1.1490 (3)	0.0203 (18)	
C2H	0.9139 (5)	0.5993 (4)	1.1339 (3)	0.0209 (19)	
C3H	0.9539 (5)	0.6116 (4)	1.0934 (3)	0.024 (2)	
C4H	0.9326 (6)	0.6627 (4)	1.0677 (3)	0.026 (2)	
C5H	0.8724 (6)	0.7001 (4)	1.0805 (3)	0.025 (2)	
C6H	0.8339 (5)	0.6860 (4)	1.1209 (3)	0.024 (2)	
P1I	0.39219 (13)	0.31893 (10)	0.61178 (7)	0.0148 (4)	
C1I	0.3701 (6)	0.2518 (4)	0.5784 (3)	0.0213 (19)	
C2I	0.4271 (6)	0.2152 (5)	0.5598 (4)	0.031 (2)	
H2I	0.4802	0.2262	0.5644	0.038*	
C3I	0.4077 (7)	0.1641 (5)	0.5349 (4)	0.042 (3)	
H3I	0.4469	0.1398	0.5217	0.050*	
C4I	0.3277 (7)	0.1471 (5)	0.5290 (3)	0.035 (3)	
H4I	0.3132	0.1113	0.5119	0.042*	
C5I	0.2724 (6)	0.1829 (5)	0.5481 (3)	0.034 (3)	
H5I	0.2195	0.1711	0.5446	0.041*	
C6I	0.2911 (6)	0.2353 (4)	0.5721 (3)	0.023 (2)	
H6I	0.2515	0.2601	0.5844	0.028*	
C7I	0.4932 (5)	0.3383 (4)	0.6067 (3)	0.0139 (16)	
C8I	0.5471 (5)	0.2992 (4)	0.6286 (3)	0.0212 (19)	
H8I	0.5302	0.2661	0.6471	0.025*	
C9I	0.6251 (5)	0.3090 (5)	0.6231 (3)	0.028 (2)	
H9I	0.6624	0.2826	0.6379	0.033*	
C10I	0.6488 (5)	0.3572 (4)	0.5961 (4)	0.026 (2)	
H10I	0.7025	0.3633	0.5915	0.031*	
C11I	0.5958 (6)	0.3959 (5)	0.5763 (3)	0.028 (2)	
H11I	0.6130	0.4297	0.5586	0.033*	
C12I	0.5162 (5)	0.3871 (4)	0.5812 (3)	0.0223 (19)	
H12I	0.4793	0.4145	0.5672	0.027*	
C13I	0.3303 (5)	0.3780 (4)	0.5895 (3)	0.0211 (19)	
C14I	0.3296 (5)	0.3919 (5)	0.5406 (3)	0.024 (2)	
H14I	0.3629	0.3706	0.5204	0.029*	
C15I	0.2801 (6)	0.4370 (6)	0.5222 (4)	0.041 (3)	
H15I	0.2805	0.4480	0.4897	0.049*	
C16I	0.2298 (7)	0.4657 (6)	0.5525 (5)	0.050 (3)	
H16I	0.1955	0.4960	0.5399	0.060*	
C17I	0.2279 (7)	0.4517 (6)	0.6001 (4)	0.045 (3)	
H17I	0.1918	0.4712	0.6196	0.054*	
C18I	0.2787 (6)	0.4093 (5)	0.6187 (4)	0.038 (3)	
H18I	0.2794	0.4008	0.6517	0.046*	
C19I	0.3772 (5)	0.3061 (4)	0.6735 (3)	0.0139 (16)	
C20I	0.3472 (5)	0.2519 (4)	0.6890 (3)	0.0182 (18)	
H20I	0.3325	0.2207	0.6671	0.022*	
C21I	0.3388 (5)	0.2437 (4)	0.7379 (3)	0.0183 (18)	
H21I	0.3193	0.2064	0.7494	0.022*	
C22I	0.3593 (5)	0.2905 (5)	0.7695 (3)	0.026 (2)	
H22I	0.3516	0.2858	0.8024	0.031*	
C23I	0.3908 (6)	0.3435 (5)	0.7527 (4)	0.029 (2)	
H23I	0.4061	0.3746	0.7745	0.035*	
C24I	0.4005 (6)	0.3522 (5)	0.7057 (3)	0.027 (2)	
H24I	0.4227	0.3888	0.6947	0.033*	
P1J	0.42376 (13)	0.06838 (10)	0.93211 (8)	0.0172 (4)	
C1J	0.4271 (5)	0.0734 (4)	0.8694 (3)	0.0168 (17)	
C2J	0.4132 (5)	0.0198 (5)	0.8435 (3)	0.026 (2)	
H2J	0.4055	−0.0178	0.8592	0.031*	
C3J	0.4111 (6)	0.0232 (6)	0.7935 (3)	0.035 (3)	
H3J	0.3975	−0.0113	0.7747	0.042*	
C4J	0.4295 (6)	0.0790 (7)	0.7722 (4)	0.045 (3)	
H4J	0.4314	0.0812	0.7387	0.054*	
C5J	0.4445 (5)	0.1299 (5)	0.7984 (4)	0.035 (3)	
H5J	0.4555	0.1670	0.7829	0.042*	
C6J	0.4439 (5)	0.1285 (4)	0.8477 (3)	0.023 (2)	
H6J	0.4547	0.1640	0.8659	0.028*	
C7J	0.4566 (5)	0.1383 (4)	0.9591 (3)	0.0222 (19)	
C8J	0.4071 (7)	0.1885 (5)	0.9574 (4)	0.034 (2)	
H8J	0.3565	0.1848	0.9429	0.040*	
C9J	0.4308 (7)	0.2431 (5)	0.9765 (4)	0.044 (3)	
H9J	0.3969	0.2771	0.9761	0.052*	
C10J	0.5073 (7)	0.2471 (5)	0.9967 (4)	0.036 (3)	
H10J	0.5249	0.2850	1.0091	0.044*	
C11J	0.5556 (6)	0.1993 (5)	0.9989 (4)	0.034 (2)	
H11J	0.6065	0.2035	1.0129	0.041*	
C12J	0.5306 (6)	0.1433 (4)	0.9804 (3)	0.023 (2)	
H12J	0.5639	0.1089	0.9824	0.028*	
C13J	0.3274 (5)	0.0540 (4)	0.9499 (3)	0.0199 (18)	
C14J	0.2718 (5)	0.0304 (4)	0.9177 (3)	0.0209 (19)	
H14J	0.2838	0.0249	0.8855	0.025*	
C15J	0.1975 (5)	0.0149 (4)	0.9330 (3)	0.0178 (18)	
H15J	0.1589	−0.0008	0.9113	0.021*	
C16J	0.1820 (5)	0.0230 (4)	0.9807 (3)	0.0232 (19)	
H16J	0.1322	0.0126	0.9915	0.028*	
C17J	0.2380 (5)	0.0460 (4)	1.0126 (3)	0.023 (2)	
H17J	0.2268	0.0506	1.0450	0.028*	
C18J	0.3105 (6)	0.0623 (5)	0.9972 (3)	0.026 (2)	
H18J	0.3484	0.0791	1.0188	0.031*	
C19J	0.4862 (5)	0.0074 (4)	0.9515 (3)	0.0202 (18)	
C20J	0.4632 (6)	−0.0339 (4)	0.9848 (3)	0.024 (2)	
H20J	0.4131	−0.0308	0.9974	0.029*	
C21J	0.5139 (7)	−0.0805 (4)	1.0000 (3)	0.032 (2)	
H21J	0.4986	−0.1097	1.0225	0.038*	
C22J	0.5880 (7)	−0.0830 (5)	0.9811 (3)	0.034 (2)	
H22J	0.6240	−0.1130	0.9923	0.040*	
C23J	0.6093 (6)	−0.0432 (4)	0.9469 (4)	0.030 (2)	
H23J	0.6587	−0.0469	0.9335	0.036*	
C24J	0.5589 (5)	0.0025 (4)	0.9318 (3)	0.024 (2)	
H24J	0.5736	0.0304	0.9081	0.028*	
P1K	0.08894 (13)	0.76538 (11)	0.21289 (8)	0.0178 (5)	
C1K	0.1611 (5)	0.8145 (4)	0.2413 (3)	0.0179 (18)	
C2K	0.2338 (5)	0.8187 (4)	0.2229 (3)	0.0200 (18)	
H2K	0.2445	0.7983	0.1943	0.024*	
C3K	0.2909 (6)	0.8528 (4)	0.2464 (3)	0.026 (2)	
H3K	0.3413	0.8555	0.2342	0.031*	
C4K	0.2739 (6)	0.8838 (4)	0.2886 (3)	0.024 (2)	
H4K	0.3128	0.9067	0.3055	0.029*	
C5K	0.1997 (6)	0.8800 (4)	0.3045 (3)	0.028 (2)	
H5K	0.1875	0.9019	0.3322	0.034*	
C6K	0.1427 (6)	0.8458 (4)	0.2821 (3)	0.025 (2)	
H6K	0.0921	0.8436	0.2939	0.029*	
C7K	0.1010 (5)	0.6905 (4)	0.2381 (3)	0.0178 (18)	
C8K	0.1352 (5)	0.6819 (4)	0.2830 (3)	0.024 (2)	
H8K	0.1555	0.7158	0.3003	0.028*	
C9K	0.1398 (5)	0.6255 (4)	0.3024 (3)	0.0223 (19)	
H9K	0.1642	0.6201	0.3329	0.027*	
C10K	0.1094 (5)	0.5762 (4)	0.2782 (3)	0.024 (2)	
H10K	0.1106	0.5373	0.2928	0.029*	
C11K	0.0771 (5)	0.5825 (4)	0.2330 (3)	0.0226 (19)	
H11K	0.0589	0.5478	0.2159	0.027*	
C12K	0.0714 (5)	0.6406 (4)	0.2124 (3)	0.0228 (19)	
H12K	0.0480	0.6459	0.1816	0.027*	
C13K	0.1026 (5)	0.7593 (4)	0.1501 (3)	0.0193 (18)	
C14K	0.1595 (5)	0.7210 (4)	0.1342 (3)	0.0238 (19)	
H14K	0.1895	0.6969	0.1561	0.029*	
C15K	0.1725 (6)	0.7180 (5)	0.0863 (3)	0.027 (2)	
H15K	0.2116	0.6917	0.0754	0.033*	
C16K	0.1300 (6)	0.7526 (4)	0.0544 (3)	0.027 (2)	
H16K	0.1403	0.7513	0.0217	0.032*	
C17K	0.0718 (5)	0.7893 (4)	0.0703 (3)	0.026 (2)	
H17K	0.0411	0.8122	0.0480	0.031*	
C18K	0.0569 (5)	0.7938 (4)	0.1181 (3)	0.0224 (19)	
H18K	0.0169	0.8195	0.1287	0.027*	
C19K	−0.0049 (5)	0.7961 (4)	0.2231 (3)	0.0176 (17)	
C20K	−0.0662 (5)	0.7575 (4)	0.2355 (3)	0.0225 (19)	
H20K	−0.0570	0.7152	0.2400	0.027*	
C21K	−0.1390 (5)	0.7809 (5)	0.2409 (3)	0.026 (2)	
H21K	−0.1805	0.7547	0.2485	0.032*	
C22K	−0.1524 (5)	0.8426 (5)	0.2353 (4)	0.029 (2)	
H22K	−0.2030	0.8587	0.2388	0.035*	
C23K	−0.0919 (6)	0.8806 (4)	0.2245 (4)	0.032 (2)	
H23K	−0.1005	0.9231	0.2217	0.038*	
C24K	−0.0193 (6)	0.8573 (4)	0.2178 (4)	0.028 (2)	
H24K	0.0215	0.8837	0.2095	0.034*	
P1L	0.12556 (13)	0.68545 (10)	0.88726 (8)	0.0172 (4)	
C1L	0.1517 (6)	0.7546 (4)	0.9187 (3)	0.025 (2)	
C2L	0.0998 (7)	0.7990 (4)	0.9281 (3)	0.031 (2)	
H2L	0.0469	0.7945	0.9177	0.037*	
C3L	0.1236 (7)	0.8518 (5)	0.9530 (4)	0.037 (3)	
H3L	0.0870	0.8826	0.9599	0.044*	
C4L	0.1999 (7)	0.8580 (5)	0.9671 (3)	0.036 (3)	
H4L	0.2160	0.8935	0.9839	0.044*	
C5L	0.2536 (7)	0.8146 (5)	0.9577 (3)	0.038 (3)	
H5L	0.3064	0.8201	0.9677	0.046*	
C6L	0.2304 (6)	0.7616 (5)	0.9330 (3)	0.027 (2)	
H6L	0.2672	0.7311	0.9262	0.01 (2)*	
C7L	0.0247 (5)	0.6711 (4)	0.8940 (3)	0.0176 (17)	
C8L	−0.0295 (6)	0.7069 (5)	0.8693 (3)	0.027 (2)	
H8L	−0.0127	0.7358	0.8470	0.033*	
C9L	−0.1070 (6)	0.7010 (5)	0.8770 (4)	0.033 (2)	
H9L	−0.1437	0.7265	0.8606	0.039*	
C10L	−0.1321 (6)	0.6575 (5)	0.9088 (4)	0.034 (2)	
H10L	−0.1857	0.6542	0.9148	0.040*	
C11L	−0.0797 (5)	0.6194 (4)	0.9313 (3)	0.025 (2)	
H11L	−0.0976	0.5879	0.9511	0.031*	
C12L	−0.0001 (5)	0.6265 (5)	0.9253 (3)	0.024 (2)	
H12L	0.0365	0.6015	0.9421	0.02 (3)*	
C13L	0.1857 (5)	0.6259 (4)	0.9115 (3)	0.0188 (18)	
C14L	0.1893 (6)	0.6162 (5)	0.9605 (3)	0.030 (2)	
H14L	0.1589	0.6404	0.9804	0.036*	
C15L	0.2373 (7)	0.5711 (5)	0.9798 (4)	0.043 (3)	
H15L	0.2404	0.5646	1.0131	0.051*	
C16L	0.2804 (7)	0.5361 (6)	0.9504 (5)	0.052 (4)	
H16L	0.3125	0.5047	0.9636	0.062*	
C17L	0.2779 (7)	0.5458 (5)	0.9023 (5)	0.043 (3)	
H17L	0.3090	0.5219	0.8826	0.051*	
C18L	0.2294 (6)	0.5910 (5)	0.8823 (4)	0.028 (2)	
H18L	0.2267	0.5974	0.8490	0.034*	
C19L	0.1375 (5)	0.6942 (4)	0.8255 (3)	0.0200 (18)	
C20L	0.1712 (6)	0.7468 (4)	0.8073 (3)	0.025 (2)	
H20L	0.1915	0.7775	0.8280	0.030*	
C21L	0.1745 (6)	0.7536 (4)	0.7582 (4)	0.030 (2)	
H21L	0.1951	0.7899	0.7455	0.036*	
C22L	0.1481 (5)	0.7083 (5)	0.7282 (3)	0.025 (2)	
H22L	0.1522	0.7132	0.6950	0.030*	
C23L	0.1155 (6)	0.6554 (5)	0.7457 (3)	0.029 (2)	
H23L	0.0975	0.6242	0.7247	0.035*	
C24L	0.1098 (6)	0.6493 (4)	0.7946 (3)	0.024 (2)	
H24L	0.0866	0.6138	0.8069	0.029*	
P1M	0.05641 (14)	0.93686 (11)	0.56977 (8)	0.0220 (5)	
C1M	0.0547 (5)	0.9384 (5)	0.6339 (3)	0.023 (2)	
C2M	0.0602 (5)	0.9943 (5)	0.6557 (3)	0.027 (2)	
H2M	0.0610	1.0303	0.6371	0.032*	
C3M	0.0645 (5)	0.9981 (6)	0.7044 (4)	0.033 (3)	
H3M	0.0681	1.0367	0.7194	0.040*	
C4M	0.0637 (6)	0.9467 (6)	0.7310 (4)	0.035 (3)	
H4M	0.0669	0.9493	0.7646	0.042*	
C5M	0.0582 (6)	0.8902 (6)	0.7089 (4)	0.034 (2)	
H5M	0.0564	0.8543	0.7275	0.041*	
C6M	0.0554 (6)	0.8861 (5)	0.6606 (4)	0.031 (2)	
H6M	0.0540	0.8475	0.6457	0.037*	
C7M	0.0265 (6)	0.8646 (5)	0.5464 (3)	0.028 (2)	
C8M	0.0779 (8)	0.8152 (5)	0.5505 (5)	0.048 (3)	
H8M	0.1278	0.8202	0.5657	0.058*	
C9M	0.0553 (8)	0.7589 (6)	0.5324 (5)	0.056 (4)	
H9M	0.0904	0.7257	0.5348	0.067*	
C10M	−0.0163 (8)	0.7506 (6)	0.5112 (5)	0.053 (4)	
H10M	−0.0314	0.7115	0.5000	0.063*	
C11M	−0.0673 (8)	0.7992 (6)	0.5059 (4)	0.049 (3)	
H11M	−0.1165	0.7940	0.4900	0.059*	
C12M	−0.0456 (6)	0.8553 (5)	0.5242 (4)	0.036 (3)	
H12M	−0.0811	0.8883	0.5215	0.044*	
C13M	0.1524 (5)	0.9524 (4)	0.5515 (3)	0.025 (2)	
C14M	0.2079 (5)	0.9785 (4)	0.5818 (3)	0.0191 (18)	
H14M	0.1967	0.9865	0.6138	0.023*	
C15M	0.2796 (5)	0.9928 (4)	0.5653 (3)	0.0226 (19)	
H15M	0.3175	1.0112	0.5861	0.027*	
C16M	0.2974 (5)	0.9811 (5)	0.5195 (3)	0.026 (2)	
H16M	0.3478	0.9899	0.5090	0.031*	
C17M	0.2406 (6)	0.9559 (5)	0.4879 (4)	0.035 (2)	
H17M	0.2519	0.9495	0.4557	0.042*	
C18M	0.1682 (6)	0.9405 (5)	0.5038 (3)	0.035 (3)	
H18M	0.1301	0.9223	0.4831	0.042*	
C19M	−0.0092 (6)	0.9938 (4)	0.5484 (3)	0.0222 (19)	
C20M	0.0078 (7)	1.0319 (5)	0.5096 (3)	0.035 (3)	
H20M	0.0558	1.0284	0.4945	0.041*	
C21M	−0.0465 (7)	1.0737 (5)	0.4944 (4)	0.038 (3)	
H21M	−0.0349	1.0995	0.4688	0.045*	
C22M	−0.1161 (7)	1.0800 (5)	0.5143 (4)	0.036 (3)	
H22M	−0.1520	1.1098	0.5027	0.043*	
C23M	−0.1349 (6)	1.0426 (5)	0.5520 (4)	0.033 (2)	
H23M	−0.1837	1.0460	0.5661	0.040*	
C24M	−0.0802 (6)	1.0000 (4)	0.5683 (4)	0.029 (2)	
H24M	−0.0923	0.9744	0.5940	0.034*	
P1N	0.40258 (14)	0.25108 (11)	1.28879 (8)	0.0211 (5)	
C1N	0.3412 (5)	0.1999 (4)	1.2568 (3)	0.0216 (19)	
C2N	0.2597 (6)	0.2032 (5)	1.2610 (3)	0.031 (2)	
H2N	0.2380	0.2304	1.2829	0.037*	
C3N	0.2120 (6)	0.1659 (5)	1.2324 (4)	0.034 (2)	
H3N	0.1573	0.1687	1.2347	0.040*	
C4N	0.2405 (6)	0.1265 (5)	1.2019 (3)	0.029 (2)	
H4N	0.2061	0.1014	1.1834	0.034*	
C5N	0.3201 (7)	0.1216 (5)	1.1971 (4)	0.036 (3)	
H5N	0.3401	0.0934	1.1753	0.043*	
C6N	0.3702 (6)	0.1581 (5)	1.2241 (3)	0.026 (2)	
H6N	0.4246	0.1549	1.2206	0.031*	
C7N	0.4024 (5)	0.3228 (4)	1.2601 (3)	0.023 (2)	
C8N	0.4579 (7)	0.3662 (5)	1.2737 (4)	0.041 (3)	
H8N	0.4976	0.3564	1.2968	0.049*	
C9N	0.4557 (7)	0.4229 (6)	1.2539 (4)	0.043 (3)	
H9N	0.4922	0.4529	1.2643	0.051*	
C10N	0.4000 (6)	0.4367 (5)	1.2184 (3)	0.028 (2)	
H10N	0.3993	0.4759	1.2043	0.033*	
C11N	0.3468 (6)	0.3948 (4)	1.2038 (3)	0.026 (2)	
H11N	0.3092	0.4048	1.1794	0.032*	
C12N	0.3464 (6)	0.3372 (4)	1.2241 (3)	0.025 (2)	
H12N	0.3088	0.3079	1.2137	0.029*	
C13N	0.3653 (6)	0.2607 (5)	1.3474 (3)	0.029 (2)	
C14N	0.3746 (6)	0.3136 (4)	1.3719 (3)	0.025 (2)	
H14N	0.3979	0.3476	1.3574	0.030*	
C15N	0.3501 (6)	0.3182 (5)	1.4180 (4)	0.036 (3)	
H15N	0.3563	0.3555	1.4347	0.043*	
C16N	0.3180 (7)	0.2704 (5)	1.4389 (4)	0.038 (3)	
H16N	0.3041	0.2735	1.4710	0.046*	
C17N	0.3046 (6)	0.2170 (5)	1.4149 (4)	0.036 (2)	
H17N	0.2787	0.1843	1.4295	0.043*	
C18N	0.3293 (6)	0.2115 (5)	1.3696 (4)	0.035 (2)	
H18N	0.3220	0.1742	1.3531	0.041*	
C19N	0.4986 (5)	0.2233 (4)	1.2934 (3)	0.026 (2)	
C20N	0.5469 (6)	0.2317 (6)	1.2563 (4)	0.045 (3)	
H20N	0.5285	0.2541	1.2294	0.054*	
C21N	0.6224 (6)	0.2079 (7)	1.2574 (4)	0.047 (3)	
H21N	0.6557	0.2146	1.2320	0.057*	
C22N	0.6473 (7)	0.1735 (6)	1.2975 (4)	0.044 (3)	
H22N	0.6974	0.1552	1.2985	0.053*	
C23N	0.6003 (6)	0.1660 (5)	1.3353 (4)	0.035 (2)	
H23N	0.6187	0.1437	1.3623	0.042*	
C24N	0.5261 (6)	0.1907 (5)	1.3342 (4)	0.033 (2)	
H24N	0.4941	0.1859	1.3605	0.039*	
O1S	0.2860 (9)	0.4190 (7)	0.8386 (5)	0.124 (5)	
H1S	0.3345	0.4203	0.8456	0.185*	
C1S	0.2488 (16)	0.3864 (13)	0.8752 (9)	0.145 (8)	
H11S	0.2002	0.3848	0.8686	0.217*	
H12S	0.2569	0.4045	0.9015	0.217*	
H13S	0.2670	0.3506	0.8771	0.217*	
O2S	0.2281 (15)	0.5108 (13)	0.7859 (6)	0.232 (9)	
H2S	0.2453	0.4822	0.8036	0.348*	
C2S	0.257 (3)	0.505 (3)	0.7458 (13)	0.260 (12)	
H21S	0.2401	0.5335	0.7276	0.390*	
H22S	0.2436	0.4710	0.7340	0.390*	
H23S	0.3060	0.5071	0.7488	0.390*	

### Atomic displacement parameters (Å^2^)

**Table:**

	*U*^11^	*U*^22^	*U*^33^	*U*^12^	*U*^13^	*U*^23^
I1	0.0303 (3)	0.0157 (3)	0.0168 (3)	0.0010 (2)	0.0020 (2)	−0.0018 (2)
I2	0.0202 (3)	0.0338 (3)	0.0161 (3)	0.0116 (3)	−0.0037 (2)	0.0005 (2)
I3	0.0193 (3)	0.0168 (3)	0.0236 (3)	0.0046 (2)	−0.0036 (2)	−0.0025 (2)
I4	0.0320 (3)	0.0160 (3)	0.0192 (3)	0.0014 (2)	0.0048 (2)	−0.0020 (2)
I5	0.0170 (3)	0.0310 (3)	0.0189 (3)	0.0064 (2)	−0.0020 (2)	0.0058 (2)
I6	0.0202 (3)	0.0154 (3)	0.0214 (3)	0.0019 (2)	−0.0011 (2)	−0.0017 (2)
I1A	0.0296 (3)	0.0223 (3)	0.0145 (3)	−0.0053 (2)	−0.0040 (2)	0.0031 (2)
I2A	0.0212 (3)	0.0237 (3)	0.0227 (3)	0.0015 (2)	−0.0021 (2)	−0.0012 (2)
I3A	0.0241 (3)	0.0243 (3)	0.0137 (3)	0.0033 (2)	−0.0057 (2)	0.0007 (2)
F1A	0.022 (3)	0.029 (3)	0.018 (2)	−0.004 (2)	−0.009 (2)	0.001 (2)
F2A	0.029 (3)	0.032 (3)	0.017 (2)	0.002 (2)	0.002 (2)	0.006 (2)
F3A	0.020 (3)	0.036 (3)	0.022 (3)	−0.008 (2)	−0.003 (2)	0.005 (2)
C1A	0.025 (5)	0.020 (5)	0.021 (4)	−0.004 (4)	−0.004 (4)	0.003 (4)
C2A	0.033 (5)	0.014 (4)	0.007 (4)	−0.005 (4)	−0.009 (3)	−0.002 (3)
C3A	0.020 (4)	0.022 (5)	0.019 (4)	−0.001 (4)	−0.004 (3)	−0.003 (3)
C4A	0.024 (4)	0.020 (4)	0.013 (4)	0.004 (4)	0.002 (3)	−0.005 (3)
C5A	0.018 (4)	0.023 (5)	0.019 (4)	0.002 (4)	−0.013 (3)	−0.005 (4)
C6A	0.027 (5)	0.013 (4)	0.022 (4)	0.002 (4)	−0.002 (4)	0.002 (3)
I1B	0.0236 (3)	0.0159 (3)	0.0233 (3)	0.0014 (2)	0.0032 (2)	−0.0047 (2)
I2B	0.0204 (3)	0.0221 (3)	0.0214 (3)	0.0045 (2)	−0.0033 (2)	−0.0011 (2)
I3B	0.0247 (3)	0.0176 (3)	0.0171 (3)	0.0023 (2)	0.0002 (2)	−0.0003 (2)
F1B	0.030 (3)	0.023 (3)	0.019 (3)	0.006 (2)	0.003 (2)	−0.002 (2)
F2B	0.028 (3)	0.018 (3)	0.023 (3)	0.011 (2)	0.001 (2)	−0.002 (2)
F3B	0.032 (3)	0.017 (3)	0.026 (3)	0.011 (2)	−0.001 (2)	0.003 (2)
C1B	0.032 (5)	0.017 (4)	0.016 (4)	0.000 (4)	0.003 (4)	−0.003 (3)
C2B	0.016 (4)	0.015 (4)	0.022 (4)	0.002 (3)	0.001 (3)	0.000 (3)
C3B	0.007 (4)	0.023 (4)	0.020 (4)	−0.002 (3)	−0.004 (3)	0.002 (3)
C4B	0.007 (4)	0.018 (4)	0.029 (5)	0.003 (3)	0.004 (3)	−0.008 (4)
C5B	0.013 (4)	0.017 (4)	0.012 (4)	−0.001 (3)	0.004 (3)	0.000 (3)
C6B	0.013 (4)	0.014 (4)	0.028 (5)	0.000 (3)	−0.003 (3)	0.005 (3)
I1C	0.0132 (2)	0.0154 (3)	0.0223 (3)	0.0010 (2)	0.0003 (2)	0.0007 (2)
I2C	0.0235 (3)	0.0142 (3)	0.0265 (3)	0.0002 (2)	−0.0016 (2)	0.0015 (2)
I3C	0.0140 (3)	0.0242 (3)	0.0231 (3)	−0.0012 (2)	−0.0030 (2)	−0.0014 (2)
F1C	0.016 (3)	0.020 (3)	0.044 (3)	−0.004 (2)	−0.008 (2)	0.003 (2)
F2C	0.017 (3)	0.021 (3)	0.028 (3)	0.009 (2)	−0.002 (2)	0.003 (2)
F3C	0.020 (3)	0.018 (3)	0.033 (3)	−0.002 (2)	0.000 (2)	0.002 (2)
C1C	0.019 (4)	0.013 (4)	0.020 (4)	−0.002 (3)	0.007 (3)	0.003 (3)
C2C	0.010 (4)	0.015 (4)	0.022 (4)	−0.001 (3)	−0.001 (3)	0.001 (3)
C3C	0.018 (4)	0.022 (5)	0.023 (5)	0.003 (4)	−0.001 (4)	0.009 (4)
C4C	0.020 (4)	0.018 (4)	0.024 (5)	0.004 (4)	−0.004 (4)	0.004 (4)
C5C	0.012 (4)	0.017 (4)	0.016 (4)	−0.001 (3)	0.003 (3)	0.000 (3)
C6C	0.024 (5)	0.017 (4)	0.020 (4)	−0.002 (4)	0.004 (4)	−0.004 (3)
I1D	0.0236 (3)	0.0177 (3)	0.0228 (3)	0.0031 (2)	0.0039 (2)	−0.0027 (2)
I2D	0.0191 (3)	0.0272 (3)	0.0224 (3)	0.0016 (2)	0.0007 (2)	−0.0070 (2)
I3D	0.0444 (4)	0.0283 (3)	0.0224 (3)	0.0031 (3)	−0.0040 (3)	0.0048 (3)
F1D	0.028 (3)	0.021 (3)	0.025 (3)	0.002 (2)	0.005 (2)	0.000 (2)
F2D	0.032 (3)	0.044 (4)	0.017 (3)	0.006 (3)	0.003 (2)	0.006 (2)
F3D	0.041 (3)	0.023 (3)	0.032 (3)	0.013 (3)	0.006 (3)	0.005 (2)
C1D	0.027 (5)	0.022 (5)	0.020 (4)	−0.009 (4)	0.001 (4)	−0.003 (4)
C2D	0.020 (4)	0.017 (4)	0.017 (4)	−0.008 (4)	−0.004 (3)	−0.003 (3)
C3D	0.019 (4)	0.024 (5)	0.024 (4)	−0.004 (4)	−0.002 (4)	−0.005 (4)
C4D	0.026 (5)	0.035 (5)	0.010 (4)	−0.005 (4)	−0.003 (3)	0.000 (4)
C5D	0.037 (6)	0.023 (5)	0.015 (4)	0.000 (4)	−0.006 (4)	0.002 (4)
C6D	0.027 (5)	0.021 (5)	0.016 (4)	0.004 (4)	−0.010 (4)	−0.007 (3)
I1E	0.0245 (3)	0.0190 (3)	0.0169 (3)	−0.0014 (2)	−0.0028 (2)	0.0031 (2)
I2E	0.0194 (3)	0.0265 (3)	0.0213 (3)	−0.0018 (2)	−0.0018 (2)	−0.0018 (2)
I3E	0.0195 (3)	0.0204 (3)	0.0166 (3)	0.0023 (2)	−0.0054 (2)	0.0000 (2)
F1E	0.020 (3)	0.028 (3)	0.018 (2)	−0.001 (2)	−0.009 (2)	0.005 (2)
F2E	0.027 (3)	0.034 (3)	0.016 (2)	0.003 (2)	−0.003 (2)	0.002 (2)
F3E	0.018 (3)	0.027 (3)	0.018 (2)	−0.003 (2)	−0.002 (2)	0.003 (2)
C1E	0.027 (5)	0.020 (5)	0.015 (4)	−0.009 (4)	−0.001 (3)	0.001 (3)
C2E	0.019 (4)	0.021 (4)	0.012 (4)	0.008 (3)	−0.005 (3)	0.006 (3)
C3E	0.025 (5)	0.016 (4)	0.016 (4)	0.006 (4)	−0.006 (3)	−0.005 (3)
C4E	0.028 (5)	0.020 (5)	0.016 (4)	0.009 (4)	−0.007 (4)	−0.003 (3)
C5E	0.028 (5)	0.021 (5)	0.014 (4)	0.011 (4)	−0.002 (4)	−0.002 (3)
C6E	0.016 (4)	0.010 (4)	0.027 (4)	−0.001 (3)	−0.003 (3)	−0.003 (3)
I1F	0.0242 (3)	0.0181 (3)	0.0251 (3)	0.0034 (2)	0.0044 (2)	−0.0045 (2)
I2F	0.0270 (3)	0.0275 (3)	0.0279 (3)	0.0109 (3)	−0.0051 (3)	−0.0034 (3)
I3F	0.0332 (3)	0.0199 (3)	0.0180 (3)	0.0018 (2)	0.0024 (2)	−0.0003 (2)
F1F	0.032 (3)	0.034 (3)	0.016 (3)	0.012 (3)	−0.005 (2)	−0.006 (2)
F2F	0.031 (3)	0.017 (3)	0.020 (3)	0.005 (2)	0.008 (2)	−0.003 (2)
F3F	0.036 (3)	0.018 (3)	0.021 (3)	0.009 (2)	0.000 (2)	0.001 (2)
C1F	0.015 (4)	0.016 (4)	0.027 (5)	−0.004 (3)	0.013 (3)	−0.003 (3)
C2F	0.017 (4)	0.020 (4)	0.021 (4)	−0.003 (3)	0.006 (3)	−0.006 (3)
C3F	0.016 (4)	0.008 (4)	0.024 (4)	0.003 (3)	0.005 (3)	−0.002 (3)
C4F	0.025 (5)	0.014 (4)	0.024 (4)	−0.003 (4)	0.012 (4)	−0.006 (3)
C5F	0.032 (5)	0.011 (4)	0.021 (4)	−0.009 (4)	0.007 (4)	−0.001 (3)
C6F	0.024 (5)	0.022 (5)	0.012 (4)	−0.003 (4)	0.006 (3)	0.003 (3)
I1G	0.0150 (3)	0.0153 (3)	0.0195 (3)	−0.0008 (2)	0.0000 (2)	0.0003 (2)
I2G	0.0212 (3)	0.0137 (3)	0.0235 (3)	−0.0001 (2)	0.0001 (2)	−0.0009 (2)
I3G	0.0160 (3)	0.0248 (3)	0.0274 (3)	−0.0037 (2)	−0.0052 (2)	0.0023 (2)
F1G	0.017 (3)	0.020 (3)	0.033 (3)	−0.005 (2)	−0.002 (2)	0.002 (2)
F2G	0.014 (2)	0.024 (3)	0.030 (3)	0.002 (2)	−0.004 (2)	0.002 (2)
F3G	0.019 (3)	0.013 (2)	0.030 (3)	−0.002 (2)	−0.004 (2)	0.006 (2)
C1G	0.010 (4)	0.025 (5)	0.016 (4)	−0.005 (3)	0.001 (3)	0.000 (3)
C2G	0.024 (5)	0.030 (5)	0.017 (4)	−0.007 (4)	−0.002 (4)	−0.007 (4)
C3G	0.021 (4)	0.008 (4)	0.017 (4)	−0.001 (3)	0.005 (3)	0.004 (3)
C4G	0.019 (4)	0.026 (5)	0.010 (4)	0.002 (4)	0.001 (3)	0.001 (3)
C5G	0.019 (4)	0.023 (5)	0.012 (4)	−0.004 (4)	0.002 (3)	−0.006 (3)
C6G	0.020 (4)	0.017 (4)	0.015 (4)	−0.002 (3)	0.004 (3)	0.002 (3)
I1H	0.0198 (3)	0.0174 (3)	0.0256 (3)	0.0007 (2)	0.0015 (2)	−0.0048 (2)
I2H	0.0175 (3)	0.0358 (4)	0.0245 (3)	0.0026 (3)	−0.0008 (2)	−0.0078 (3)
I3H	0.0485 (4)	0.0378 (4)	0.0357 (4)	0.0079 (3)	−0.0110 (3)	0.0115 (3)
F1H	0.025 (3)	0.019 (3)	0.028 (3)	0.005 (2)	0.000 (2)	0.001 (2)
F2H	0.042 (4)	0.045 (4)	0.021 (3)	−0.004 (3)	0.007 (3)	0.002 (3)
F3H	0.034 (3)	0.029 (3)	0.037 (3)	0.014 (3)	0.004 (3)	0.004 (3)
C1H	0.023 (5)	0.017 (4)	0.020 (4)	0.006 (4)	−0.007 (4)	−0.001 (3)
C2H	0.020 (4)	0.012 (4)	0.030 (5)	−0.001 (3)	−0.007 (4)	−0.006 (3)
C3H	0.025 (5)	0.023 (5)	0.022 (4)	0.002 (4)	−0.004 (4)	−0.017 (4)
C4H	0.035 (5)	0.023 (5)	0.020 (5)	−0.012 (4)	−0.008 (4)	0.003 (4)
C5H	0.030 (5)	0.023 (5)	0.022 (5)	0.003 (4)	−0.009 (4)	−0.001 (4)
C6H	0.024 (5)	0.017 (5)	0.031 (5)	0.008 (4)	0.002 (4)	−0.005 (4)
P1I	0.0177 (11)	0.0133 (10)	0.0134 (10)	−0.0001 (8)	0.0006 (8)	0.0015 (8)
C1I	0.033 (5)	0.011 (4)	0.020 (4)	−0.002 (4)	0.004 (4)	−0.006 (3)
C2I	0.030 (5)	0.027 (5)	0.037 (6)	−0.003 (4)	0.005 (4)	−0.003 (4)
C3I	0.058 (8)	0.025 (6)	0.044 (7)	0.001 (5)	0.016 (6)	−0.009 (5)
C4I	0.067 (8)	0.019 (5)	0.021 (5)	−0.014 (5)	0.014 (5)	−0.003 (4)
C5I	0.040 (6)	0.040 (6)	0.023 (5)	−0.023 (5)	0.011 (4)	−0.009 (4)
C6I	0.029 (5)	0.025 (5)	0.017 (4)	−0.003 (4)	0.011 (4)	−0.002 (4)
C7I	0.016 (4)	0.013 (4)	0.014 (4)	−0.007 (3)	0.007 (3)	0.001 (3)
C8I	0.017 (4)	0.025 (5)	0.021 (4)	0.002 (4)	−0.001 (3)	0.013 (4)
C9I	0.018 (5)	0.038 (6)	0.028 (5)	0.012 (4)	0.001 (4)	0.012 (4)
C10I	0.013 (4)	0.025 (5)	0.039 (6)	0.007 (4)	0.005 (4)	0.001 (4)
C11I	0.031 (5)	0.027 (5)	0.026 (5)	0.000 (4)	0.014 (4)	0.005 (4)
C12I	0.023 (5)	0.016 (4)	0.028 (5)	0.009 (4)	−0.002 (4)	0.005 (4)
C13I	0.015 (4)	0.025 (5)	0.023 (4)	−0.011 (4)	0.000 (3)	0.006 (4)
C14I	0.024 (5)	0.032 (5)	0.015 (4)	−0.004 (4)	−0.010 (4)	0.007 (4)
C15I	0.038 (6)	0.057 (8)	0.025 (5)	0.010 (6)	−0.018 (5)	0.011 (5)
C16I	0.031 (6)	0.043 (7)	0.075 (9)	0.014 (5)	−0.004 (6)	0.022 (7)
C17I	0.043 (7)	0.040 (7)	0.051 (7)	0.024 (6)	0.006 (6)	0.008 (6)
C18I	0.025 (5)	0.048 (7)	0.043 (6)	0.005 (5)	0.011 (5)	0.005 (5)
C19I	0.013 (4)	0.019 (4)	0.009 (4)	−0.002 (3)	−0.001 (3)	−0.001 (3)
C20I	0.015 (4)	0.017 (4)	0.022 (4)	−0.002 (3)	−0.002 (3)	0.000 (3)
C21I	0.013 (4)	0.022 (4)	0.020 (4)	−0.004 (3)	−0.003 (3)	0.001 (3)
C22I	0.022 (5)	0.039 (6)	0.017 (4)	0.007 (4)	0.000 (4)	0.000 (4)
C23I	0.026 (5)	0.032 (6)	0.028 (5)	−0.012 (4)	−0.006 (4)	−0.005 (4)
C24I	0.031 (5)	0.030 (5)	0.021 (5)	−0.014 (4)	0.001 (4)	−0.003 (4)
P1J	0.0199 (11)	0.0157 (11)	0.0160 (10)	−0.0009 (9)	−0.0001 (8)	−0.0028 (8)
C1J	0.013 (4)	0.017 (4)	0.021 (4)	0.004 (3)	0.001 (3)	0.006 (3)
C2J	0.028 (5)	0.029 (5)	0.021 (5)	−0.008 (4)	−0.001 (4)	−0.004 (4)
C3J	0.031 (5)	0.055 (7)	0.019 (5)	−0.008 (5)	0.006 (4)	−0.018 (5)
C4J	0.021 (5)	0.097 (11)	0.018 (5)	−0.016 (6)	−0.001 (4)	0.006 (6)
C5J	0.013 (4)	0.042 (6)	0.048 (6)	−0.006 (4)	−0.006 (4)	0.018 (5)
C6J	0.017 (4)	0.019 (5)	0.034 (5)	0.005 (4)	0.004 (4)	0.008 (4)
C7J	0.024 (5)	0.021 (5)	0.022 (4)	−0.002 (4)	0.001 (4)	−0.007 (4)
C8J	0.040 (6)	0.029 (6)	0.031 (5)	0.012 (5)	−0.003 (5)	−0.007 (4)
C9J	0.056 (8)	0.026 (6)	0.049 (7)	−0.003 (5)	0.007 (6)	−0.012 (5)
C10J	0.057 (7)	0.024 (5)	0.030 (5)	−0.021 (5)	0.013 (5)	−0.014 (4)
C11J	0.031 (6)	0.039 (6)	0.033 (6)	−0.018 (5)	0.006 (4)	−0.004 (5)
C12J	0.035 (5)	0.018 (5)	0.016 (4)	−0.011 (4)	0.001 (4)	−0.003 (3)
C13J	0.022 (4)	0.016 (4)	0.022 (4)	0.010 (4)	0.006 (3)	0.005 (3)
C14J	0.023 (5)	0.016 (4)	0.023 (4)	−0.003 (4)	0.006 (4)	−0.005 (4)
C15J	0.015 (4)	0.018 (4)	0.021 (4)	0.012 (3)	0.002 (3)	−0.003 (3)
C16J	0.022 (5)	0.021 (5)	0.027 (5)	0.007 (4)	0.005 (4)	0.006 (4)
C17J	0.028 (5)	0.030 (5)	0.014 (4)	0.008 (4)	0.013 (4)	0.003 (4)
C18J	0.026 (5)	0.032 (5)	0.021 (4)	0.005 (4)	0.004 (4)	−0.005 (4)
C19J	0.022 (4)	0.021 (4)	0.017 (4)	−0.002 (4)	−0.003 (3)	−0.003 (3)
C20J	0.036 (5)	0.023 (5)	0.013 (4)	0.002 (4)	0.003 (4)	−0.002 (3)
C21J	0.063 (7)	0.021 (5)	0.012 (4)	0.011 (5)	0.002 (4)	0.002 (4)
C22J	0.055 (7)	0.023 (5)	0.022 (5)	0.014 (5)	−0.009 (5)	−0.009 (4)
C23J	0.025 (5)	0.020 (5)	0.045 (6)	0.002 (4)	0.001 (4)	0.001 (4)
C24J	0.026 (5)	0.022 (5)	0.023 (5)	−0.001 (4)	0.003 (4)	0.004 (4)
P1K	0.0176 (11)	0.0175 (11)	0.0182 (11)	−0.0015 (9)	0.0006 (9)	−0.0006 (8)
C1K	0.017 (4)	0.022 (5)	0.015 (4)	−0.001 (3)	0.002 (3)	0.004 (3)
C2K	0.020 (4)	0.019 (4)	0.020 (4)	−0.007 (4)	−0.003 (3)	−0.003 (3)
C3K	0.027 (5)	0.019 (5)	0.033 (5)	−0.002 (4)	0.006 (4)	0.007 (4)
C4K	0.031 (5)	0.015 (4)	0.027 (5)	−0.007 (4)	−0.004 (4)	0.004 (4)
C5K	0.044 (6)	0.018 (5)	0.023 (5)	−0.006 (4)	0.007 (4)	−0.009 (4)
C6K	0.027 (5)	0.022 (5)	0.026 (5)	−0.009 (4)	0.005 (4)	−0.003 (4)
C7K	0.020 (4)	0.022 (5)	0.012 (4)	−0.001 (4)	0.005 (3)	−0.008 (3)
C8K	0.022 (5)	0.028 (5)	0.020 (4)	−0.004 (4)	−0.006 (4)	−0.006 (4)
C9K	0.020 (4)	0.029 (5)	0.017 (4)	0.008 (4)	−0.004 (3)	0.001 (4)
C10K	0.022 (5)	0.019 (5)	0.031 (5)	0.011 (4)	0.001 (4)	0.005 (4)
C11K	0.025 (5)	0.016 (4)	0.026 (5)	−0.001 (4)	−0.003 (4)	−0.003 (4)
C12K	0.023 (5)	0.019 (4)	0.026 (5)	−0.002 (4)	−0.013 (4)	−0.002 (4)
C13K	0.026 (5)	0.019 (4)	0.013 (4)	0.003 (4)	0.001 (3)	−0.001 (3)
C14K	0.023 (5)	0.023 (5)	0.024 (5)	0.005 (4)	−0.003 (4)	−0.004 (4)
C15K	0.030 (5)	0.030 (5)	0.022 (5)	0.009 (4)	0.004 (4)	0.000 (4)
C16K	0.041 (6)	0.023 (5)	0.016 (4)	−0.003 (4)	−0.007 (4)	0.003 (4)
C17K	0.026 (5)	0.026 (5)	0.025 (5)	0.003 (4)	0.002 (4)	0.005 (4)
C18K	0.021 (4)	0.025 (5)	0.021 (4)	0.002 (4)	−0.004 (4)	−0.002 (4)
C19K	0.010 (4)	0.027 (5)	0.017 (4)	0.004 (3)	0.009 (3)	−0.005 (3)
C20K	0.021 (4)	0.019 (4)	0.028 (5)	−0.004 (4)	−0.001 (4)	−0.003 (4)
C21K	0.016 (4)	0.029 (5)	0.034 (5)	−0.004 (4)	0.003 (4)	0.002 (4)
C22K	0.012 (4)	0.027 (5)	0.048 (6)	0.002 (4)	0.005 (4)	−0.004 (5)
C23K	0.030 (5)	0.019 (5)	0.046 (6)	0.003 (4)	0.002 (5)	0.000 (4)
C24K	0.026 (5)	0.018 (5)	0.041 (6)	−0.010 (4)	0.004 (4)	−0.003 (4)
P1L	0.0206 (11)	0.0158 (11)	0.0154 (10)	−0.0026 (9)	0.0016 (8)	0.0015 (8)
C1L	0.045 (6)	0.020 (5)	0.010 (4)	0.000 (4)	0.007 (4)	0.002 (3)
C2L	0.047 (6)	0.021 (5)	0.024 (5)	0.004 (5)	−0.003 (4)	0.005 (4)
C3L	0.063 (8)	0.019 (5)	0.028 (5)	0.006 (5)	0.009 (5)	−0.001 (4)
C4L	0.063 (8)	0.031 (6)	0.017 (5)	−0.024 (5)	0.016 (5)	−0.009 (4)
C5L	0.056 (7)	0.040 (6)	0.019 (5)	−0.031 (6)	0.014 (5)	−0.018 (4)
C6L	0.031 (5)	0.028 (5)	0.022 (5)	−0.015 (4)	0.010 (4)	−0.007 (4)
C7L	0.018 (4)	0.021 (4)	0.014 (4)	0.000 (3)	0.004 (3)	−0.003 (3)
C8L	0.032 (5)	0.030 (5)	0.020 (5)	−0.001 (4)	0.000 (4)	0.006 (4)
C9L	0.028 (5)	0.035 (6)	0.034 (6)	0.006 (5)	−0.002 (4)	−0.001 (5)
C10L	0.030 (6)	0.040 (6)	0.031 (6)	−0.004 (5)	0.004 (4)	0.001 (5)
C11L	0.020 (5)	0.024 (5)	0.033 (5)	−0.010 (4)	0.006 (4)	0.006 (4)
C12L	0.018 (4)	0.030 (5)	0.025 (5)	−0.003 (4)	0.005 (4)	0.004 (4)
C13L	0.019 (4)	0.018 (4)	0.019 (4)	−0.003 (3)	−0.001 (3)	0.008 (3)
C14L	0.036 (6)	0.030 (5)	0.023 (5)	−0.004 (5)	−0.002 (4)	0.005 (4)
C15L	0.043 (7)	0.036 (7)	0.049 (7)	−0.001 (5)	−0.007 (5)	0.025 (5)
C16L	0.042 (7)	0.035 (7)	0.076 (9)	0.003 (6)	−0.016 (7)	0.025 (7)
C17L	0.032 (6)	0.029 (6)	0.066 (8)	0.006 (5)	0.003 (6)	0.012 (6)
C18L	0.023 (5)	0.028 (5)	0.035 (5)	−0.011 (4)	0.005 (4)	0.003 (4)
C19L	0.022 (4)	0.023 (5)	0.015 (4)	0.000 (4)	0.003 (3)	0.004 (3)
C20L	0.028 (5)	0.021 (5)	0.025 (5)	−0.008 (4)	−0.004 (4)	−0.002 (4)
C21L	0.040 (6)	0.019 (5)	0.032 (5)	−0.007 (4)	0.011 (4)	0.010 (4)
C22L	0.017 (4)	0.038 (6)	0.020 (4)	−0.002 (4)	0.007 (4)	0.004 (4)
C23L	0.035 (6)	0.033 (6)	0.019 (5)	−0.012 (5)	0.003 (4)	−0.004 (4)
C24L	0.032 (5)	0.022 (5)	0.018 (4)	−0.006 (4)	−0.002 (4)	0.004 (4)
P1M	0.0212 (11)	0.0208 (12)	0.0242 (12)	−0.0038 (9)	0.0041 (9)	−0.0078 (9)
C1M	0.015 (4)	0.034 (5)	0.020 (4)	−0.003 (4)	0.000 (3)	−0.005 (4)
C2M	0.026 (5)	0.034 (6)	0.021 (5)	−0.005 (4)	0.012 (4)	−0.007 (4)
C3M	0.011 (4)	0.055 (7)	0.032 (5)	−0.015 (4)	0.003 (4)	−0.013 (5)
C4M	0.020 (5)	0.064 (8)	0.022 (5)	0.008 (5)	−0.004 (4)	0.011 (5)
C5M	0.017 (5)	0.051 (7)	0.035 (6)	0.000 (5)	−0.001 (4)	0.017 (5)
C6M	0.021 (5)	0.032 (6)	0.040 (6)	−0.001 (4)	0.003 (4)	0.000 (5)
C7M	0.029 (5)	0.027 (5)	0.029 (5)	−0.012 (4)	0.008 (4)	−0.009 (4)
C8M	0.051 (8)	0.037 (7)	0.058 (8)	−0.013 (6)	0.027 (6)	−0.009 (6)
C9M	0.064 (9)	0.027 (6)	0.080 (10)	−0.005 (6)	0.040 (8)	−0.005 (6)
C10M	0.068 (9)	0.029 (7)	0.062 (8)	−0.017 (6)	0.020 (7)	−0.022 (6)
C11M	0.060 (8)	0.042 (7)	0.047 (7)	−0.025 (6)	0.006 (6)	−0.016 (6)
C12M	0.034 (6)	0.036 (6)	0.040 (6)	−0.013 (5)	0.011 (5)	−0.007 (5)
C13M	0.027 (5)	0.027 (5)	0.023 (5)	−0.010 (4)	0.012 (4)	−0.007 (4)
C14M	0.027 (5)	0.016 (4)	0.015 (4)	0.000 (4)	−0.004 (3)	0.005 (3)
C15M	0.021 (5)	0.017 (4)	0.029 (5)	0.006 (4)	−0.004 (4)	−0.001 (4)
C16M	0.016 (4)	0.034 (5)	0.029 (5)	0.002 (4)	0.005 (4)	−0.002 (4)
C17M	0.035 (6)	0.043 (6)	0.028 (5)	0.000 (5)	0.010 (4)	−0.014 (5)
C18M	0.025 (5)	0.051 (7)	0.028 (5)	−0.009 (5)	0.006 (4)	−0.019 (5)
C19M	0.034 (5)	0.014 (4)	0.018 (4)	−0.005 (4)	0.001 (4)	−0.001 (3)
C20M	0.054 (7)	0.034 (6)	0.017 (5)	−0.003 (5)	0.015 (5)	−0.009 (4)
C21M	0.071 (8)	0.021 (5)	0.022 (5)	0.004 (5)	0.011 (5)	0.000 (4)
C22M	0.054 (7)	0.025 (5)	0.029 (5)	0.012 (5)	−0.008 (5)	−0.009 (4)
C23M	0.025 (5)	0.038 (6)	0.037 (6)	0.002 (4)	−0.004 (4)	−0.006 (5)
C24M	0.035 (6)	0.020 (5)	0.031 (5)	−0.011 (4)	0.002 (4)	0.003 (4)
P1N	0.0208 (12)	0.0232 (12)	0.0192 (11)	−0.0055 (10)	−0.0010 (9)	0.0045 (9)
C1N	0.015 (4)	0.028 (5)	0.021 (4)	−0.003 (4)	0.001 (3)	−0.005 (4)
C2N	0.027 (5)	0.037 (6)	0.029 (5)	0.002 (4)	0.006 (4)	−0.008 (4)
C3N	0.025 (5)	0.033 (6)	0.042 (6)	−0.006 (4)	−0.003 (5)	−0.003 (5)
C4N	0.039 (6)	0.023 (5)	0.023 (5)	−0.008 (4)	0.001 (4)	0.003 (4)
C5N	0.056 (7)	0.022 (5)	0.032 (5)	0.000 (5)	0.005 (5)	0.004 (4)
C6N	0.019 (5)	0.036 (6)	0.022 (5)	−0.003 (4)	0.005 (4)	0.004 (4)
C7N	0.026 (5)	0.023 (5)	0.021 (4)	−0.004 (4)	0.007 (4)	0.005 (4)
C8N	0.036 (6)	0.040 (6)	0.044 (7)	−0.005 (5)	−0.016 (5)	0.009 (5)
C9N	0.043 (7)	0.040 (7)	0.044 (6)	−0.016 (5)	−0.011 (5)	0.012 (5)
C10N	0.032 (5)	0.028 (5)	0.023 (5)	−0.001 (4)	0.002 (4)	0.001 (4)
C11N	0.028 (5)	0.025 (5)	0.026 (5)	−0.003 (4)	−0.001 (4)	0.005 (4)
C12N	0.025 (5)	0.023 (5)	0.026 (5)	0.001 (4)	−0.003 (4)	−0.002 (4)
C13N	0.033 (5)	0.025 (5)	0.028 (5)	−0.010 (4)	−0.005 (4)	0.007 (4)
C14N	0.031 (5)	0.026 (5)	0.019 (4)	−0.009 (4)	−0.005 (4)	0.010 (4)
C15N	0.047 (7)	0.031 (6)	0.030 (5)	−0.009 (5)	−0.001 (5)	0.000 (4)
C16N	0.050 (7)	0.043 (7)	0.021 (5)	−0.017 (5)	0.004 (5)	−0.006 (4)
C17N	0.032 (6)	0.037 (6)	0.038 (6)	−0.009 (5)	0.004 (5)	0.004 (5)
C18N	0.049 (7)	0.025 (5)	0.030 (5)	−0.011 (5)	0.003 (5)	−0.008 (4)
C19N	0.022 (5)	0.024 (5)	0.031 (5)	−0.001 (4)	−0.004 (4)	0.002 (4)
C20N	0.033 (6)	0.066 (9)	0.034 (6)	0.017 (6)	−0.005 (5)	0.017 (6)
C21N	0.027 (6)	0.094 (11)	0.021 (5)	0.005 (6)	0.001 (4)	0.016 (6)
C22N	0.037 (6)	0.050 (7)	0.046 (7)	0.013 (6)	0.006 (5)	0.013 (6)
C23N	0.032 (6)	0.040 (6)	0.032 (6)	0.007 (5)	0.005 (4)	0.007 (5)
C24N	0.028 (5)	0.036 (6)	0.035 (5)	−0.011 (4)	0.005 (4)	0.007 (5)
O1S	0.129 (12)	0.131 (12)	0.112 (11)	−0.015 (10)	0.021	−0.001 (10)
C1S	0.160 (17)	0.146 (17)	0.132 (16)	−0.020 (15)	0.052 (13)	−0.002 (15)
O2S	0.31 (3)	0.28 (2)	0.111 (13)	0.07 (2)	0.070	0.035
C2S	0.33 (3)	0.30 (3)	0.15 (2)	0.08 (2)	0.092 (17)	0.061 (17)

### Geometric parameters (Å, º)

**Table:**

I1A—C1A	2.093 (9)	C1K—C6K	1.390 (12)
I2A—C3A	2.104 (9)	C2K—C3K	1.385 (13)
I3A—C5A	2.099 (8)	C2K—H2K	0.9500
F1A—C2A	1.339 (9)	C3K—C4K	1.414 (13)
F2A—C4A	1.347 (10)	C3K—H3K	0.9500
F3A—C6A	1.364 (10)	C4K—C5K	1.378 (14)
C1A—C6A	1.375 (12)	C4K—H4K	0.9500
C1A—C2A	1.410 (13)	C5K—C6K	1.370 (13)
C2A—C3A	1.380 (12)	C5K—H5K	0.9500
C3A—C4A	1.387 (12)	C6K—H6K	0.9500
C4A—C5A	1.377 (12)	C7K—C8K	1.390 (12)
C5A—C6A	1.379 (12)	C7K—C12K	1.399 (12)
I1B—C1B	2.100 (9)	C8K—C9K	1.358 (13)
I2B—C3B	2.090 (8)	C8K—H8K	0.9500
I3B—C5B	2.106 (8)	C9K—C10K	1.373 (13)
F1B—C2B	1.341 (10)	C9K—H9K	0.9500
F2B—C4B	1.357 (9)	C10K—C11K	1.377 (13)
F3B—C6B	1.357 (10)	C10K—H10K	0.9500
C1B—C2B	1.376 (12)	C11K—C12K	1.407 (13)
C1B—C6B	1.385 (12)	C11K—H11K	0.9500
C2B—C3B	1.398 (12)	C12K—H12K	0.9500
C3B—C4B	1.380 (12)	C13K—C14K	1.384 (12)
C4B—C5B	1.383 (12)	C13K—C18K	1.395 (12)
C5B—C6B	1.372 (12)	C14K—C15K	1.383 (12)
I1C—C1C	2.131 (9)	C14K—H14K	0.9500
I2C—C3C	2.089 (10)	C15K—C16K	1.368 (13)
I3C—C5C	2.088 (8)	C15K—H15K	0.9500
F1C—C2C	1.351 (9)	C16K—C17K	1.377 (13)
F2C—C4C	1.356 (10)	C16K—H16K	0.9500
F3C—C6C	1.326 (10)	C17K—C18K	1.389 (12)
C1C—C6C	1.381 (12)	C17K—H17K	0.9500
C1C—C2C	1.405 (12)	C18K—H18K	0.9500
C2C—C3C	1.384 (12)	C19K—C24K	1.375 (13)
C3C—C4C	1.359 (13)	C19K—C20K	1.411 (12)
C4C—C5C	1.400 (12)	C20K—C21K	1.372 (13)
C5C—C6C	1.387 (12)	C20K—H20K	0.9500
I1D—C1D	2.082 (9)	C21K—C22K	1.385 (14)
I2D—C3D	2.112 (9)	C21K—H21K	0.9500
I3D—C5D	2.104 (9)	C22K—C23K	1.381 (13)
F1D—C2D	1.341 (10)	C22K—H22K	0.9500
F2D—C4D	1.338 (10)	C23K—C24K	1.373 (13)
F3D—C6D	1.350 (10)	C23K—H23K	0.9500
C1D—C2D	1.397 (13)	C24K—H24K	0.9500
C1D—C6D	1.405 (13)	P1L—C19L	1.777 (9)
C2D—C3D	1.363 (12)	P1L—C7L	1.784 (9)
C3D—C4D	1.395 (13)	P1L—C13L	1.788 (9)
C4D—C5D	1.369 (14)	P1L—C1L	1.809 (10)
C5D—C6D	1.382 (13)	C1L—C2L	1.359 (14)
I1E—C1E	2.101 (8)	C1L—C6L	1.407 (14)
I2E—C3E	2.113 (9)	C2L—C3L	1.410 (14)
I3E—C5E	2.112 (9)	C2L—H2L	0.9500
F1E—C2E	1.365 (9)	C3L—C4L	1.364 (16)
F2E—C4E	1.350 (10)	C3L—H3L	0.9500
F3E—C6E	1.339 (10)	C4L—C5L	1.364 (17)
C1E—C2E	1.371 (12)	C4L—H4L	0.9500
C1E—C6E	1.393 (12)	C5L—C6L	1.407 (13)
C2E—C3E	1.367 (12)	C5L—H5L	0.9500
C3E—C4E	1.364 (12)	C6L—H6L	0.9500
C4E—C5E	1.395 (13)	C7L—C8L	1.386 (13)
C5E—C6E	1.378 (12)	C7L—C12L	1.400 (12)
I1F—C1F	2.127 (9)	C8L—C9L	1.369 (14)
I2F—C3F	2.088 (8)	C8L—H8L	0.9500
I3F—C5F	2.090 (9)	C9L—C10L	1.395 (15)
F1F—C2F	1.352 (10)	C9L—H9L	0.9500
F2F—C4F	1.338 (10)	C10L—C11L	1.368 (14)
F3F—C6F	1.348 (10)	C10L—H10L	0.9500
C1F—C6F	1.377 (12)	C11L—C12L	1.399 (12)
C1F—C2F	1.378 (13)	C11L—H11L	0.9500
C2F—C3F	1.383 (11)	C12L—H12L	0.9500
C3F—C4F	1.383 (12)	C13L—C18L	1.376 (13)
C4F—C5F	1.381 (13)	C13L—C14L	1.398 (12)
C5F—C6F	1.383 (12)	C14L—C15L	1.389 (14)
I1G—C1G	2.117 (9)	C14L—H14L	0.9500
I2G—C3G	2.106 (8)	C15L—C16L	1.375 (18)
I3G—C5G	2.082 (9)	C15L—H15L	0.9500
F1G—C2G	1.359 (10)	C16L—C17L	1.376 (17)
F2G—C4G	1.347 (10)	C16L—H16L	0.9500
F3G—C6G	1.357 (10)	C17L—C18L	1.401 (15)
C1G—C6G	1.382 (11)	C17L—H17L	0.9500
C1G—C2G	1.384 (13)	C18L—H18L	0.9500
C2G—C3G	1.373 (13)	C19L—C24L	1.391 (13)
C3G—C4G	1.382 (12)	C19L—C20L	1.403 (13)
C4G—C5G	1.386 (13)	C20L—C21L	1.398 (13)
C5G—C6G	1.397 (12)	C20L—H20L	0.9500
I1H—C1H	2.116 (9)	C21L—C22L	1.373 (14)
I2H—C3H	2.092 (9)	C21L—H21L	0.9500
I3H—C5H	2.106 (9)	C22L—C23L	1.392 (14)
F1H—C2H	1.332 (10)	C22L—H22L	0.9500
F2H—C4H	1.344 (11)	C23L—C24L	1.397 (12)
F3H—C6H	1.354 (10)	C23L—H23L	0.9500
C1H—C6H	1.383 (13)	C24L—H24L	0.9500
C1H—C2H	1.400 (12)	P1M—C19M	1.776 (10)
C2H—C3H	1.387 (13)	P1M—C13M	1.787 (9)
C3H—C4H	1.381 (13)	P1M—C7M	1.790 (10)
C4H—C5H	1.385 (14)	P1M—C1M	1.815 (9)
C5H—C6H	1.379 (13)	C1M—C6M	1.375 (14)
P1I—C13I	1.779 (10)	C1M—C2M	1.377 (13)
P1I—C1I	1.784 (9)	C2M—C3M	1.378 (13)
P1I—C19I	1.798 (8)	C2M—H2M	0.9500
P1I—C7I	1.804 (8)	C3M—C4M	1.358 (16)
C1I—C2I	1.390 (13)	C3M—H3M	0.9500
C1I—C6I	1.414 (13)	C4M—C5M	1.392 (17)
C2I—C3I	1.360 (14)	C4M—H4M	0.9500
C2I—H2I	0.9500	C5M—C6M	1.365 (14)
C3I—C4I	1.432 (16)	C5M—H5M	0.9500
C3I—H3I	0.9500	C6M—H6M	0.9500
C4I—C5I	1.365 (15)	C7M—C12M	1.383 (14)
C4I—H4I	0.9500	C7M—C8M	1.402 (16)
C5I—C6I	1.370 (13)	C8M—C9M	1.390 (17)
C5I—H5I	0.9500	C8M—H8M	0.9500
C6I—H6I	0.9500	C9M—C10M	1.36 (2)
C7I—C12I	1.363 (12)	C9M—H9M	0.9500
C7I—C8I	1.390 (12)	C10M—C11M	1.389 (19)
C8I—C9I	1.376 (12)	C10M—H10M	0.9500
C8I—H8I	0.9500	C11M—C12M	1.383 (15)
C9I—C10I	1.379 (14)	C11M—H11M	0.9500
C9I—H9I	0.9500	C12M—H12M	0.9500
C10I—C11I	1.352 (13)	C13M—C14M	1.380 (12)
C10I—H10I	0.9500	C13M—C18M	1.411 (12)
C11I—C12I	1.398 (13)	C14M—C15M	1.377 (13)
C11I—H11I	0.9500	C14M—H14M	0.9500
C12I—H12I	0.9500	C15M—C16M	1.368 (13)
C13I—C14I	1.414 (12)	C15M—H15M	0.9500
C13I—C18I	1.418 (14)	C16M—C17M	1.409 (14)
C14I—C15I	1.395 (14)	C16M—H16M	0.9500
C14I—H14I	0.9500	C17M—C18M	1.387 (14)
C15I—C16I	1.395 (17)	C17M—H17M	0.9500
C15I—H15I	0.9500	C18M—H18M	0.9500
C16I—C17I	1.381 (17)	C19M—C24M	1.376 (13)
C16I—H16I	0.9500	C19M—C20M	1.420 (13)
C17I—C18I	1.370 (15)	C20M—C21M	1.368 (15)
C17I—H17I	0.9500	C20M—H20M	0.9500
C18I—H18I	0.9500	C21M—C22M	1.353 (16)
C19I—C20I	1.379 (12)	C21M—H21M	0.9500
C19I—C24I	1.408 (12)	C22M—C23M	1.394 (15)
C20I—C21I	1.406 (12)	C22M—H22M	0.9500
C20I—H20I	0.9500	C23M—C24M	1.394 (14)
C21I—C22I	1.399 (13)	C23M—H23M	0.9500
C21I—H21I	0.9500	C24M—H24M	0.9500
C22I—C23I	1.379 (14)	P1N—C19N	1.764 (10)
C22I—H22I	0.9500	P1N—C1N	1.767 (9)
C23I—C24I	1.361 (13)	P1N—C7N	1.773 (10)
C23I—H23I	0.9500	P1N—C13N	1.815 (10)
C24I—H24I	0.9500	C1N—C6N	1.411 (13)
P1J—C1J	1.779 (9)	C1N—C2N	1.415 (13)
P1J—C13J	1.785 (9)	C2N—C3N	1.395 (14)
P1J—C19J	1.792 (9)	C2N—H2N	0.9500
P1J—C7J	1.798 (9)	C3N—C4N	1.331 (14)
C1J—C6J	1.395 (12)	C3N—H3N	0.9500
C1J—C2J	1.403 (13)	C4N—C5N	1.390 (15)
C2J—C3J	1.414 (13)	C4N—H4N	0.9500
C2J—H2J	0.9500	C5N—C6N	1.384 (14)
C3J—C4J	1.411 (17)	C5N—H5N	0.9500
C3J—H3J	0.9500	C6N—H6N	0.9500
C4J—C5J	1.361 (17)	C7N—C8N	1.395 (14)
C4J—H4J	0.9500	C7N—C12N	1.409 (13)
C5J—C6J	1.393 (14)	C8N—C9N	1.367 (16)
C5J—H5J	0.9500	C8N—H8N	0.9500
C6J—H6J	0.9500	C9N—C10N	1.391 (14)
C7J—C12J	1.390 (13)	C9N—H9N	0.9500
C7J—C8J	1.395 (14)	C10N—C11N	1.352 (14)
C8J—C9J	1.372 (15)	C10N—H10N	0.9500
C8J—H8J	0.9500	C11N—C12N	1.392 (13)
C9J—C10J	1.416 (17)	C11N—H11N	0.9500
C9J—H9J	0.9500	C12N—H12N	0.9500
C10J—C11J	1.342 (16)	C13N—C14N	1.359 (14)
C10J—H10J	0.9500	C13N—C18N	1.410 (14)
C11J—C12J	1.399 (14)	C14N—C15N	1.390 (14)
C11J—H11J	0.9500	C14N—H14N	0.9500
C12J—H12J	0.9500	C15N—C16N	1.340 (15)
C13J—C18J	1.391 (12)	C15N—H15N	0.9500
C13J—C14J	1.393 (13)	C16N—C17N	1.369 (15)
C14J—C15J	1.410 (12)	C16N—H16N	0.9500
C14J—H14J	0.9500	C17N—C18N	1.373 (14)
C15J—C16J	1.397 (12)	C17N—H17N	0.9500
C15J—H15J	0.9500	C18N—H18N	0.9500
C16J—C17J	1.387 (13)	C19N—C20N	1.377 (15)
C16J—H16J	0.9500	C19N—C24N	1.421 (13)
C17J—C18J	1.388 (13)	C20N—C21N	1.401 (15)
C17J—H17J	0.9500	C20N—H20N	0.9500
C18J—H18J	0.9500	C21N—C22N	1.415 (15)
C19J—C20J	1.377 (12)	C21N—H21N	0.9500
C19J—C24J	1.398 (13)	C22N—C23N	1.378 (15)
C20J—C21J	1.403 (13)	C22N—H22N	0.9500
C20J—H20J	0.9500	C23N—C24N	1.388 (14)
C21J—C22J	1.406 (15)	C23N—H23N	0.9500
C21J—H21J	0.9500	C24N—H24N	0.9500
C22J—C23J	1.367 (14)	O1S—C1S	1.43 (3)
C22J—H22J	0.9500	O1S—H1S	0.850 (15)
C23J—C24J	1.384 (13)	C1S—H11S	0.85 (3)
C23J—H23J	0.9500	C1S—H12S	0.85 (3)
C24J—H24J	0.9500	C1S—H13S	0.85 (3)
P1K—C19K	1.788 (8)	O2S—C2S	1.26 (3)
P1K—C7K	1.802 (10)	O2S—H2S	0.85 (2)
P1K—C13K	1.805 (8)	C2S—H21S	0.85 (5)
P1K—C1K	1.810 (9)	C2S—H22S	0.85 (6)
C1K—C2K	1.380 (12)	C2S—H23S	0.85 (5)
			
C6A—C1A—C2A	115.3 (8)	C2K—C3K—H3K	120.1
C6A—C1A—I1A	124.8 (7)	C4K—C3K—H3K	120.1
C2A—C1A—I1A	119.9 (6)	C5K—C4K—C3K	118.4 (9)
F1A—C2A—C3A	118.6 (8)	C5K—C4K—H4K	120.8
F1A—C2A—C1A	118.0 (7)	C3K—C4K—H4K	120.8
C3A—C2A—C1A	123.4 (8)	C6K—C5K—C4K	122.6 (9)
C2A—C3A—C4A	116.9 (8)	C6K—C5K—H5K	118.7
C2A—C3A—I2A	120.7 (6)	C4K—C5K—H5K	118.7
C4A—C3A—I2A	122.3 (7)	C5K—C6K—C1K	118.1 (9)
F2A—C4A—C5A	118.3 (8)	C5K—C6K—H6K	121.0
F2A—C4A—C3A	118.8 (8)	C1K—C6K—H6K	121.0
C5A—C4A—C3A	122.8 (8)	C8K—C7K—C12K	119.8 (9)
C4A—C5A—C6A	117.1 (8)	C8K—C7K—P1K	121.5 (7)
C4A—C5A—I3A	120.7 (7)	C12K—C7K—P1K	118.6 (7)
C6A—C5A—I3A	122.2 (7)	C9K—C8K—C7K	120.6 (9)
F3A—C6A—C1A	116.6 (8)	C9K—C8K—H8K	119.7
F3A—C6A—C5A	119.0 (8)	C7K—C8K—H8K	119.7
C1A—C6A—C5A	124.3 (9)	C8K—C9K—C10K	120.4 (8)
C2B—C1B—C6B	117.0 (8)	C8K—C9K—H9K	119.8
C2B—C1B—I1B	122.4 (6)	C10K—C9K—H9K	119.8
C6B—C1B—I1B	120.6 (7)	C9K—C10K—C11K	120.8 (9)
F1B—C2B—C1B	119.4 (8)	C9K—C10K—H10K	119.6
F1B—C2B—C3B	118.2 (8)	C11K—C10K—H10K	119.6
C1B—C2B—C3B	122.4 (8)	C10K—C11K—C12K	119.6 (8)
C4B—C3B—C2B	116.8 (8)	C10K—C11K—H11K	120.2
C4B—C3B—I2B	121.5 (7)	C12K—C11K—H11K	120.2
C2B—C3B—I2B	121.7 (6)	C7K—C12K—C11K	118.8 (8)
F2B—C4B—C3B	118.2 (8)	C7K—C12K—H12K	120.6
F2B—C4B—C5B	118.1 (8)	C11K—C12K—H12K	120.6
C3B—C4B—C5B	123.7 (8)	C14K—C13K—C18K	120.6 (8)
C6B—C5B—C4B	116.2 (8)	C14K—C13K—P1K	119.2 (7)
C6B—C5B—I3B	122.5 (6)	C18K—C13K—P1K	120.2 (7)
C4B—C5B—I3B	121.3 (6)	C15K—C14K—C13K	119.6 (8)
F3B—C6B—C5B	118.0 (8)	C15K—C14K—H14K	120.2
F3B—C6B—C1B	118.0 (8)	C13K—C14K—H14K	120.2
C5B—C6B—C1B	124.0 (8)	C16K—C15K—C14K	120.9 (9)
C6C—C1C—C2C	117.2 (8)	C16K—C15K—H15K	119.5
C6C—C1C—I1C	120.6 (6)	C14K—C15K—H15K	119.5
C2C—C1C—I1C	122.2 (6)	C15K—C16K—C17K	119.1 (9)
F1C—C2C—C3C	118.8 (8)	C15K—C16K—H16K	120.4
F1C—C2C—C1C	118.4 (7)	C17K—C16K—H16K	120.4
C3C—C2C—C1C	122.8 (8)	C16K—C17K—C18K	121.8 (9)
C4C—C3C—C2C	116.8 (9)	C16K—C17K—H17K	119.1
C4C—C3C—I2C	120.7 (7)	C18K—C17K—H17K	119.1
C2C—C3C—I2C	122.5 (7)	C17K—C18K—C13K	117.9 (8)
F2C—C4C—C3C	119.6 (8)	C17K—C18K—H18K	121.0
F2C—C4C—C5C	116.3 (8)	C13K—C18K—H18K	121.0
C3C—C4C—C5C	124.0 (8)	C24K—C19K—C20K	119.0 (8)
C6C—C5C—C4C	116.6 (8)	C24K—C19K—P1K	120.8 (7)
C6C—C5C—I3C	120.1 (6)	C20K—C19K—P1K	120.3 (7)
C4C—C5C—I3C	123.3 (6)	C21K—C20K—C19K	119.9 (9)
F3C—C6C—C1C	118.2 (8)	C21K—C20K—H20K	120.0
F3C—C6C—C5C	119.4 (8)	C19K—C20K—H20K	120.0
C1C—C6C—C5C	122.4 (8)	C20K—C21K—C22K	120.3 (9)
C2D—C1D—C6D	114.3 (8)	C20K—C21K—H21K	119.8
C2D—C1D—I1D	120.5 (7)	C22K—C21K—H21K	119.8
C6D—C1D—I1D	125.1 (7)	C23K—C22K—C21K	119.6 (9)
F1D—C2D—C3D	118.4 (8)	C23K—C22K—H22K	120.2
F1D—C2D—C1D	117.7 (8)	C21K—C22K—H22K	120.2
C3D—C2D—C1D	123.9 (9)	C24K—C23K—C22K	120.4 (9)
C2D—C3D—C4D	118.4 (9)	C24K—C23K—H23K	119.8
C2D—C3D—I2D	120.3 (7)	C22K—C23K—H23K	119.8
C4D—C3D—I2D	121.2 (7)	C23K—C24K—C19K	120.7 (9)
F2D—C4D—C5D	119.2 (9)	C23K—C24K—H24K	119.6
F2D—C4D—C3D	119.3 (9)	C19K—C24K—H24K	119.6
C5D—C4D—C3D	121.5 (8)	C19L—P1L—C7L	106.2 (4)
C4D—C5D—C6D	117.7 (9)	C19L—P1L—C13L	111.5 (4)
C4D—C5D—I3D	120.3 (7)	C7L—P1L—C13L	112.4 (4)
C6D—C5D—I3D	122.0 (7)	C19L—P1L—C1L	110.7 (4)
F3D—C6D—C5D	119.1 (8)	C7L—P1L—C1L	108.6 (4)
F3D—C6D—C1D	116.8 (8)	C13L—P1L—C1L	107.5 (4)
C5D—C6D—C1D	124.1 (9)	C2L—C1L—C6L	120.0 (9)
C2E—C1E—C6E	116.1 (8)	C2L—C1L—P1L	123.3 (8)
C2E—C1E—I1E	121.8 (6)	C6L—C1L—P1L	116.7 (7)
C6E—C1E—I1E	122.0 (7)	C1L—C2L—C3L	120.7 (11)
F1E—C2E—C3E	117.8 (8)	C1L—C2L—H2L	119.7
F1E—C2E—C1E	117.4 (7)	C3L—C2L—H2L	119.7
C3E—C2E—C1E	124.6 (8)	C4L—C3L—C2L	119.0 (10)
C4E—C3E—C2E	116.7 (9)	C4L—C3L—H3L	120.5
C4E—C3E—I2E	122.3 (7)	C2L—C3L—H3L	120.5
C2E—C3E—I2E	121.0 (6)	C3L—C4L—C5L	121.8 (10)
F2E—C4E—C3E	119.2 (8)	C3L—C4L—H4L	119.1
F2E—C4E—C5E	118.0 (7)	C5L—C4L—H4L	119.1
C3E—C4E—C5E	122.8 (9)	C4L—C5L—C6L	119.8 (11)
C6E—C5E—C4E	117.2 (8)	C4L—C5L—H5L	120.1
C6E—C5E—I3E	122.0 (7)	C6L—C5L—H5L	120.1
C4E—C5E—I3E	120.8 (6)	C5L—C6L—C1L	118.9 (10)
F3E—C6E—C5E	119.1 (8)	C5L—C6L—H6L	120.6
F3E—C6E—C1E	118.6 (8)	C1L—C6L—H6L	120.6
C5E—C6E—C1E	122.4 (8)	C8L—C7L—C12L	119.8 (8)
C6F—C1F—C2F	118.9 (8)	C8L—C7L—P1L	119.0 (7)
C6F—C1F—I1F	119.3 (7)	C12L—C7L—P1L	121.1 (7)
C2F—C1F—I1F	121.8 (6)	C9L—C8L—C7L	120.6 (9)
F1F—C2F—C1F	119.3 (8)	C9L—C8L—H8L	119.7
F1F—C2F—C3F	119.0 (8)	C7L—C8L—H8L	119.7
C1F—C2F—C3F	121.6 (8)	C8L—C9L—C10L	119.8 (10)
C4F—C3F—C2F	117.0 (8)	C8L—C9L—H9L	120.1
C4F—C3F—I2F	120.8 (6)	C10L—C9L—H9L	120.1
C2F—C3F—I2F	122.2 (7)	C11L—C10L—C9L	120.3 (10)
F2F—C4F—C5F	117.9 (8)	C11L—C10L—H10L	119.9
F2F—C4F—C3F	118.5 (8)	C9L—C10L—H10L	119.9
C5F—C4F—C3F	123.6 (8)	C10L—C11L—C12L	120.4 (9)
C4F—C5F—C6F	116.7 (8)	C10L—C11L—H11L	119.8
C4F—C5F—I3F	120.2 (6)	C12L—C11L—H11L	119.8
C6F—C5F—I3F	123.0 (7)	C11L—C12L—C7L	118.9 (9)
F3F—C6F—C1F	118.9 (8)	C11L—C12L—H12L	120.5
F3F—C6F—C5F	119.0 (8)	C7L—C12L—H12L	120.5
C1F—C6F—C5F	122.1 (9)	C18L—C13L—C14L	120.4 (9)
C6G—C1G—C2G	115.6 (8)	C18L—C13L—P1L	120.1 (7)
C6G—C1G—I1G	120.8 (7)	C14L—C13L—P1L	119.4 (7)
C2G—C1G—I1G	123.6 (6)	C15L—C14L—C13L	120.0 (10)
F1G—C2G—C3G	118.6 (8)	C15L—C14L—H14L	120.0
F1G—C2G—C1G	117.4 (8)	C13L—C14L—H14L	120.0
C3G—C2G—C1G	124.1 (8)	C16L—C15L—C14L	119.4 (11)
C2G—C3G—C4G	117.2 (8)	C16L—C15L—H15L	120.3
C2G—C3G—I2G	121.5 (7)	C14L—C15L—H15L	120.3
C4G—C3G—I2G	121.2 (6)	C15L—C16L—C17L	121.0 (11)
F2G—C4G—C3G	119.5 (8)	C15L—C16L—H16L	119.5
F2G—C4G—C5G	117.6 (8)	C17L—C16L—H16L	119.5
C3G—C4G—C5G	122.9 (8)	C16L—C17L—C18L	120.1 (12)
C4G—C5G—C6G	116.0 (8)	C16L—C17L—H17L	119.9
C4G—C5G—I3G	123.4 (7)	C18L—C17L—H17L	119.9
C6G—C5G—I3G	120.4 (6)	C13L—C18L—C17L	119.1 (10)
F3G—C6G—C1G	117.9 (8)	C13L—C18L—H18L	120.4
F3G—C6G—C5G	117.9 (7)	C17L—C18L—H18L	120.4
C1G—C6G—C5G	124.2 (8)	C24L—C19L—C20L	119.5 (8)
C6H—C1H—C2H	117.0 (8)	C24L—C19L—P1L	119.2 (7)
C6H—C1H—I1H	123.8 (7)	C20L—C19L—P1L	121.2 (7)
C2H—C1H—I1H	119.2 (7)	C21L—C20L—C19L	119.2 (9)
F1H—C2H—C3H	119.1 (8)	C21L—C20L—H20L	120.4
F1H—C2H—C1H	118.4 (8)	C19L—C20L—H20L	120.4
C3H—C2H—C1H	122.5 (9)	C22L—C21L—C20L	120.5 (9)
C4H—C3H—C2H	117.6 (9)	C22L—C21L—H21L	119.7
C4H—C3H—I2H	123.3 (7)	C20L—C21L—H21L	119.7
C2H—C3H—I2H	119.0 (7)	C21L—C22L—C23L	121.1 (9)
F2H—C4H—C3H	118.5 (9)	C21L—C22L—H22L	119.5
F2H—C4H—C5H	119.4 (9)	C23L—C22L—H22L	119.5
C3H—C4H—C5H	122.1 (9)	C22L—C23L—C24L	118.6 (9)
C6H—C5H—C4H	118.4 (9)	C22L—C23L—H23L	120.7
C6H—C5H—I3H	122.1 (7)	C24L—C23L—H23L	120.7
C4H—C5H—I3H	119.5 (7)	C19L—C24L—C23L	121.1 (9)
F3H—C6H—C5H	118.6 (8)	C19L—C24L—H24L	119.5
F3H—C6H—C1H	119.0 (8)	C23L—C24L—H24L	119.5
C5H—C6H—C1H	122.4 (9)	C19M—P1M—C13M	110.5 (5)
C13I—P1I—C1I	107.9 (4)	C19M—P1M—C7M	109.5 (5)
C13I—P1I—C19I	110.4 (4)	C13M—P1M—C7M	108.5 (5)
C1I—P1I—C19I	110.3 (4)	C19M—P1M—C1M	106.8 (4)
C13I—P1I—C7I	111.4 (4)	C13M—P1M—C1M	109.9 (4)
C1I—P1I—C7I	109.7 (4)	C7M—P1M—C1M	111.6 (5)
C19I—P1I—C7I	107.1 (4)	C6M—C1M—C2M	120.2 (9)
C2I—C1I—C6I	119.7 (8)	C6M—C1M—P1M	122.2 (8)
C2I—C1I—P1I	122.8 (8)	C2M—C1M—P1M	117.3 (8)
C6I—C1I—P1I	117.4 (7)	C1M—C2M—C3M	119.9 (10)
C3I—C2I—C1I	120.9 (10)	C1M—C2M—H2M	120.0
C3I—C2I—H2I	119.6	C3M—C2M—H2M	120.0
C1I—C2I—H2I	119.6	C4M—C3M—C2M	120.1 (11)
C2I—C3I—C4I	119.3 (10)	C4M—C3M—H3M	119.9
C2I—C3I—H3I	120.3	C2M—C3M—H3M	119.9
C4I—C3I—H3I	120.3	C3M—C4M—C5M	119.8 (9)
C5I—C4I—C3I	119.2 (9)	C3M—C4M—H4M	120.1
C5I—C4I—H4I	120.4	C5M—C4M—H4M	120.1
C3I—C4I—H4I	120.4	C6M—C5M—C4M	120.4 (10)
C4I—C5I—C6I	122.0 (10)	C6M—C5M—H5M	119.8
C4I—C5I—H5I	119.0	C4M—C5M—H5M	119.8
C6I—C5I—H5I	119.0	C5M—C6M—C1M	119.5 (10)
C5I—C6I—C1I	118.8 (9)	C5M—C6M—H6M	120.3
C5I—C6I—H6I	120.6	C1M—C6M—H6M	120.3
C1I—C6I—H6I	120.6	C12M—C7M—C8M	118.5 (10)
C12I—C7I—C8I	121.2 (8)	C12M—C7M—P1M	122.4 (9)
C12I—C7I—P1I	122.2 (7)	C8M—C7M—P1M	119.1 (8)
C8I—C7I—P1I	116.5 (6)	C9M—C8M—C7M	119.5 (13)
C9I—C8I—C7I	119.3 (8)	C9M—C8M—H8M	120.2
C9I—C8I—H8I	120.3	C7M—C8M—H8M	120.2
C7I—C8I—H8I	120.3	C10M—C9M—C8M	121.1 (13)
C8I—C9I—C10I	119.8 (9)	C10M—C9M—H9M	119.4
C8I—C9I—H9I	120.1	C8M—C9M—H9M	119.4
C10I—C9I—H9I	120.1	C9M—C10M—C11M	120.1 (11)
C11I—C10I—C9I	120.2 (9)	C9M—C10M—H10M	119.9
C11I—C10I—H10I	119.9	C11M—C10M—H10M	119.9
C9I—C10I—H10I	119.9	C12M—C11M—C10M	119.2 (12)
C10I—C11I—C12I	121.3 (9)	C12M—C11M—H11M	120.4
C10I—C11I—H11I	119.3	C10M—C11M—H11M	120.4
C12I—C11I—H11I	119.3	C7M—C12M—C11M	121.5 (12)
C7I—C12I—C11I	118.1 (8)	C7M—C12M—H12M	119.2
C7I—C12I—H12I	120.9	C11M—C12M—H12M	119.2
C11I—C12I—H12I	120.9	C14M—C13M—C18M	120.8 (9)
C14I—C13I—C18I	119.0 (9)	C14M—C13M—P1M	121.7 (7)
C14I—C13I—P1I	118.9 (7)	C18M—C13M—P1M	117.3 (7)
C18I—C13I—P1I	122.0 (7)	C15M—C14M—C13M	119.5 (8)
C15I—C14I—C13I	119.8 (10)	C15M—C14M—H14M	120.2
C15I—C14I—H14I	120.1	C13M—C14M—H14M	120.2
C13I—C14I—H14I	120.1	C16M—C15M—C14M	121.3 (9)
C16I—C15I—C14I	118.6 (10)	C16M—C15M—H15M	119.4
C16I—C15I—H15I	120.7	C14M—C15M—H15M	119.4
C14I—C15I—H15I	120.7	C15M—C16M—C17M	119.7 (9)
C17I—C16I—C15I	122.6 (10)	C15M—C16M—H16M	120.1
C17I—C16I—H16I	118.7	C17M—C16M—H16M	120.1
C15I—C16I—H16I	118.7	C18M—C17M—C16M	120.0 (9)
C18I—C17I—C16I	118.9 (11)	C18M—C17M—H17M	120.0
C18I—C17I—H17I	120.5	C16M—C17M—H17M	120.0
C16I—C17I—H17I	120.5	C17M—C18M—C13M	118.6 (9)
C17I—C18I—C13I	120.9 (11)	C17M—C18M—H18M	120.7
C17I—C18I—H18I	119.5	C13M—C18M—H18M	120.7
C13I—C18I—H18I	119.5	C24M—C19M—C20M	118.3 (9)
C20I—C19I—C24I	121.3 (8)	C24M—C19M—P1M	119.6 (7)
C20I—C19I—P1I	121.0 (6)	C20M—C19M—P1M	122.0 (8)
C24I—C19I—P1I	117.6 (7)	C21M—C20M—C19M	118.5 (10)
C19I—C20I—C21I	118.6 (8)	C21M—C20M—H20M	120.7
C19I—C20I—H20I	120.7	C19M—C20M—H20M	120.7
C21I—C20I—H20I	120.7	C22M—C21M—C20M	123.1 (10)
C22I—C21I—C20I	120.0 (8)	C22M—C21M—H21M	118.5
C22I—C21I—H21I	120.0	C20M—C21M—H21M	118.5
C20I—C21I—H21I	120.0	C21M—C22M—C23M	119.7 (10)
C23I—C22I—C21I	119.7 (9)	C21M—C22M—H22M	120.1
C23I—C22I—H22I	120.2	C23M—C22M—H22M	120.1
C21I—C22I—H22I	120.2	C22M—C23M—C24M	118.3 (10)
C24I—C23I—C22I	121.4 (9)	C22M—C23M—H23M	120.9
C24I—C23I—H23I	119.3	C24M—C23M—H23M	120.9
C22I—C23I—H23I	119.3	C19M—C24M—C23M	122.1 (9)
C23I—C24I—C19I	119.0 (9)	C19M—C24M—H24M	119.0
C23I—C24I—H24I	120.5	C23M—C24M—H24M	119.0
C19I—C24I—H24I	120.5	C19N—P1N—C1N	110.8 (5)
C1J—P1J—C13J	111.3 (4)	C19N—P1N—C7N	109.0 (5)
C1J—P1J—C19J	107.9 (4)	C1N—P1N—C7N	110.1 (5)
C13J—P1J—C19J	109.5 (4)	C19N—P1N—C13N	110.0 (5)
C1J—P1J—C7J	110.3 (4)	C1N—P1N—C13N	108.3 (4)
C13J—P1J—C7J	108.2 (4)	C7N—P1N—C13N	108.6 (5)
C19J—P1J—C7J	109.7 (4)	C6N—C1N—C2N	118.0 (9)
C6J—C1J—C2J	122.4 (8)	C6N—C1N—P1N	121.9 (7)
C6J—C1J—P1J	120.6 (7)	C2N—C1N—P1N	119.9 (7)
C2J—C1J—P1J	117.0 (7)	C3N—C2N—C1N	118.9 (9)
C1J—C2J—C3J	118.2 (9)	C3N—C2N—H2N	120.6
C1J—C2J—H2J	120.9	C1N—C2N—H2N	120.6
C3J—C2J—H2J	120.9	C4N—C3N—C2N	122.2 (10)
C4J—C3J—C2J	118.5 (10)	C4N—C3N—H3N	118.9
C4J—C3J—H3J	120.8	C2N—C3N—H3N	118.9
C2J—C3J—H3J	120.8	C3N—C4N—C5N	120.6 (10)
C5J—C4J—C3J	121.6 (9)	C3N—C4N—H4N	119.7
C5J—C4J—H4J	119.2	C5N—C4N—H4N	119.7
C3J—C4J—H4J	119.2	C6N—C5N—C4N	119.7 (10)
C4J—C5J—C6J	121.1 (10)	C6N—C5N—H5N	120.2
C4J—C5J—H5J	119.4	C4N—C5N—H5N	120.2
C6J—C5J—H5J	119.4	C5N—C6N—C1N	120.6 (9)
C5J—C6J—C1J	118.0 (10)	C5N—C6N—H6N	119.7
C5J—C6J—H6J	121.0	C1N—C6N—H6N	119.7
C1J—C6J—H6J	121.0	C8N—C7N—C12N	119.0 (9)
C12J—C7J—C8J	120.1 (9)	C8N—C7N—P1N	119.8 (8)
C12J—C7J—P1J	121.1 (7)	C12N—C7N—P1N	121.2 (7)
C8J—C7J—P1J	118.8 (7)	C9N—C8N—C7N	120.4 (10)
C9J—C8J—C7J	120.5 (11)	C9N—C8N—H8N	119.8
C9J—C8J—H8J	119.8	C7N—C8N—H8N	119.8
C7J—C8J—H8J	119.8	C8N—C9N—C10N	120.0 (10)
C8J—C9J—C10J	118.1 (11)	C8N—C9N—H9N	120.0
C8J—C9J—H9J	121.0	C10N—C9N—H9N	120.0
C10J—C9J—H9J	121.0	C11N—C10N—C9N	120.6 (10)
C11J—C10J—C9J	122.3 (10)	C11N—C10N—H10N	119.7
C11J—C10J—H10J	118.8	C9N—C10N—H10N	119.7
C9J—C10J—H10J	118.8	C10N—C11N—C12N	120.8 (9)
C10J—C11J—C12J	119.5 (10)	C10N—C11N—H11N	119.6
C10J—C11J—H11J	120.3	C12N—C11N—H11N	119.6
C12J—C11J—H11J	120.3	C11N—C12N—C7N	119.2 (9)
C7J—C12J—C11J	119.5 (10)	C11N—C12N—H12N	120.4
C7J—C12J—H12J	120.2	C7N—C12N—H12N	120.4
C11J—C12J—H12J	120.2	C14N—C13N—C18N	118.4 (9)
C18J—C13J—C14J	120.7 (8)	C14N—C13N—P1N	121.8 (7)
C18J—C13J—P1J	118.9 (7)	C18N—C13N—P1N	119.8 (8)
C14J—C13J—P1J	120.2 (7)	C13N—C14N—C15N	120.4 (9)
C13J—C14J—C15J	119.8 (8)	C13N—C14N—H14N	119.8
C13J—C14J—H14J	120.1	C15N—C14N—H14N	119.8
C15J—C14J—H14J	120.1	C16N—C15N—C14N	120.2 (10)
C16J—C15J—C14J	118.7 (8)	C16N—C15N—H15N	119.9
C16J—C15J—H15J	120.7	C14N—C15N—H15N	119.9
C14J—C15J—H15J	120.7	C15N—C16N—C17N	121.3 (10)
C17J—C16J—C15J	121.0 (9)	C15N—C16N—H16N	119.3
C17J—C16J—H16J	119.5	C17N—C16N—H16N	119.3
C15J—C16J—H16J	119.5	C16N—C17N—C18N	119.0 (10)
C16J—C17J—C18J	120.1 (8)	C16N—C17N—H17N	120.5
C16J—C17J—H17J	119.9	C18N—C17N—H17N	120.5
C18J—C17J—H17J	119.9	C17N—C18N—C13N	120.5 (10)
C17J—C18J—C13J	119.7 (9)	C17N—C18N—H18N	119.8
C17J—C18J—H18J	120.2	C13N—C18N—H18N	119.8
C13J—C18J—H18J	120.2	C20N—C19N—C24N	119.6 (9)
C20J—C19J—C24J	120.8 (9)	C20N—C19N—P1N	119.5 (8)
C20J—C19J—P1J	121.1 (7)	C24N—C19N—P1N	120.9 (8)
C24J—C19J—P1J	118.1 (7)	C19N—C20N—C21N	121.6 (10)
C19J—C20J—C21J	119.8 (9)	C19N—C20N—H20N	119.2
C19J—C20J—H20J	120.1	C21N—C20N—H20N	119.2
C21J—C20J—H20J	120.1	C20N—C21N—C22N	117.8 (10)
C20J—C21J—C22J	118.4 (9)	C20N—C21N—H21N	121.1
C20J—C21J—H21J	120.8	C22N—C21N—H21N	121.1
C22J—C21J—H21J	120.8	C23N—C22N—C21N	121.1 (11)
C23J—C22J—C21J	121.3 (9)	C23N—C22N—H22N	119.5
C23J—C22J—H22J	119.3	C21N—C22N—H22N	119.5
C21J—C22J—H22J	119.3	C22N—C23N—C24N	120.5 (10)
C22J—C23J—C24J	120.0 (10)	C22N—C23N—H23N	119.7
C22J—C23J—H23J	120.0	C24N—C23N—H23N	119.7
C24J—C23J—H23J	120.0	C23N—C24N—C19N	119.3 (9)
C23J—C24J—C19J	119.6 (9)	C23N—C24N—H24N	120.3
C23J—C24J—H24J	120.2	C19N—C24N—H24N	120.3
C19J—C24J—H24J	120.2	C1S—O1S—H1S	108.6 (18)
C19K—P1K—C7K	111.8 (4)	O1S—C1S—H11S	109 (3)
C19K—P1K—C13K	110.1 (4)	O1S—C1S—H12S	109 (3)
C7K—P1K—C13K	107.6 (4)	H11S—C1S—H12S	109 (3)
C19K—P1K—C1K	108.2 (4)	O1S—C1S—H13S	109 (2)
C7K—P1K—C1K	107.7 (4)	H11S—C1S—H13S	109 (3)
C13K—P1K—C1K	111.4 (4)	H12S—C1S—H13S	110 (3)
C2K—C1K—C6K	121.5 (8)	C2S—O2S—H2S	108 (3)
C2K—C1K—P1K	119.3 (7)	O2S—C2S—H21S	109 (4)
C6K—C1K—P1K	119.1 (7)	O2S—C2S—H22S	110 (4)
C1K—C2K—C3K	119.5 (8)	H21S—C2S—H22S	110 (5)
C1K—C2K—H2K	120.2	O2S—C2S—H23S	109 (4)
C3K—C2K—H2K	120.2	H21S—C2S—H23S	109 (5)
C2K—C3K—C4K	119.8 (9)	H22S—C2S—H23S	109 (5)
			
C6A—C1A—C2A—F1A	177.4 (8)	C19J—P1J—C7J—C12J	15.9 (9)
I1A—C1A—C2A—F1A	−4.1 (11)	C1J—P1J—C7J—C8J	75.6 (9)
C6A—C1A—C2A—C3A	−1.2 (14)	C13J—P1J—C7J—C8J	−46.3 (9)
I1A—C1A—C2A—C3A	177.4 (7)	C19J—P1J—C7J—C8J	−165.7 (8)
F1A—C2A—C3A—C4A	−178.9 (8)	C12J—C7J—C8J—C9J	0.2 (16)
C1A—C2A—C3A—C4A	−0.4 (14)	P1J—C7J—C8J—C9J	−178.2 (9)
F1A—C2A—C3A—I2A	4.0 (11)	C7J—C8J—C9J—C10J	1.6 (17)
C1A—C2A—C3A—I2A	−177.5 (7)	C8J—C9J—C10J—C11J	−2.0 (17)
C2A—C3A—C4A—F2A	179.7 (8)	C9J—C10J—C11J—C12J	0.5 (16)
I2A—C3A—C4A—F2A	−3.2 (12)	C8J—C7J—C12J—C11J	−1.7 (14)
C2A—C3A—C4A—C5A	3.0 (14)	P1J—C7J—C12J—C11J	176.7 (7)
I2A—C3A—C4A—C5A	−179.9 (7)	C10J—C11J—C12J—C7J	1.4 (15)
F2A—C4A—C5A—C6A	179.4 (8)	C1J—P1J—C13J—C18J	−165.5 (7)
C3A—C4A—C5A—C6A	−3.9 (14)	C19J—P1J—C13J—C18J	75.3 (8)
F2A—C4A—C5A—I3A	−0.7 (12)	C7J—P1J—C13J—C18J	−44.2 (9)
C3A—C4A—C5A—I3A	176.0 (7)	C1J—P1J—C13J—C14J	19.2 (9)
C2A—C1A—C6A—F3A	178.6 (8)	C19J—P1J—C13J—C14J	−100.0 (8)
I1A—C1A—C6A—F3A	0.1 (12)	C7J—P1J—C13J—C14J	140.5 (7)
C2A—C1A—C6A—C5A	0.2 (14)	C18J—C13J—C14J—C15J	0.0 (13)
I1A—C1A—C6A—C5A	−178.2 (7)	P1J—C13J—C14J—C15J	175.2 (7)
C4A—C5A—C6A—F3A	−176.1 (8)	C13J—C14J—C15J—C16J	−0.7 (13)
I3A—C5A—C6A—F3A	4.1 (12)	C14J—C15J—C16J—C17J	0.2 (13)
C4A—C5A—C6A—C1A	2.2 (14)	C15J—C16J—C17J—C18J	1.0 (14)
I3A—C5A—C6A—C1A	−177.7 (7)	C16J—C17J—C18J—C13J	−1.7 (14)
C6B—C1B—C2B—F1B	−177.5 (8)	C14J—C13J—C18J—C17J	1.2 (14)
I1B—C1B—C2B—F1B	0.9 (12)	P1J—C13J—C18J—C17J	−174.1 (7)
C6B—C1B—C2B—C3B	3.3 (14)	C1J—P1J—C19J—C20J	−135.8 (7)
I1B—C1B—C2B—C3B	−178.3 (7)	C13J—P1J—C19J—C20J	−14.5 (9)
F1B—C2B—C3B—C4B	177.8 (7)	C7J—P1J—C19J—C20J	104.0 (8)
C1B—C2B—C3B—C4B	−3.1 (13)	C1J—P1J—C19J—C24J	43.7 (8)
F1B—C2B—C3B—I2B	−4.2 (11)	C13J—P1J—C19J—C24J	165.0 (7)
C1B—C2B—C3B—I2B	175.0 (7)	C7J—P1J—C19J—C24J	−76.4 (8)
C2B—C3B—C4B—F2B	−178.2 (7)	C24J—C19J—C20J—C21J	1.7 (14)
I2B—C3B—C4B—F2B	3.8 (11)	P1J—C19J—C20J—C21J	−178.8 (7)
C2B—C3B—C4B—C5B	0.7 (13)	C19J—C20J—C21J—C22J	1.0 (14)
I2B—C3B—C4B—C5B	−177.3 (6)	C20J—C21J—C22J—C23J	−3.4 (15)
F2B—C4B—C5B—C6B	−180.0 (7)	C21J—C22J—C23J—C24J	3.0 (15)
C3B—C4B—C5B—C6B	1.1 (13)	C22J—C23J—C24J—C19J	−0.3 (15)
F2B—C4B—C5B—I3B	0.8 (11)	C20J—C19J—C24J—C23J	−2.0 (14)
C3B—C4B—C5B—I3B	−178.1 (6)	P1J—C19J—C24J—C23J	178.4 (7)
C4B—C5B—C6B—F3B	179.5 (7)	C19K—P1K—C1K—C2K	−151.0 (7)
I3B—C5B—C6B—F3B	−1.2 (11)	C7K—P1K—C1K—C2K	87.9 (8)
C4B—C5B—C6B—C1B	−0.9 (13)	C13K—P1K—C1K—C2K	−29.9 (9)
I3B—C5B—C6B—C1B	178.4 (7)	C19K—P1K—C1K—C6K	30.7 (9)
C2B—C1B—C6B—F3B	178.4 (8)	C7K—P1K—C1K—C6K	−90.4 (8)
I1B—C1B—C6B—F3B	−0.1 (11)	C13K—P1K—C1K—C6K	151.8 (7)
C2B—C1B—C6B—C5B	−1.3 (14)	C6K—C1K—C2K—C3K	2.8 (14)
I1B—C1B—C6B—C5B	−179.7 (7)	P1K—C1K—C2K—C3K	−175.5 (7)
C6C—C1C—C2C—F1C	−179.0 (7)	C1K—C2K—C3K—C4K	−1.0 (13)
I1C—C1C—C2C—F1C	0.9 (11)	C2K—C3K—C4K—C5K	−1.4 (14)
C6C—C1C—C2C—C3C	1.8 (13)	C3K—C4K—C5K—C6K	2.2 (15)
I1C—C1C—C2C—C3C	−178.3 (7)	C4K—C5K—C6K—C1K	−0.6 (15)
F1C—C2C—C3C—C4C	178.9 (8)	C2K—C1K—C6K—C5K	−2.0 (14)
C1C—C2C—C3C—C4C	−1.9 (13)	P1K—C1K—C6K—C5K	176.3 (7)
F1C—C2C—C3C—I2C	−2.6 (11)	C19K—P1K—C7K—C8K	−94.3 (8)
C1C—C2C—C3C—I2C	176.6 (7)	C13K—P1K—C7K—C8K	144.6 (7)
C2C—C3C—C4C—F2C	179.2 (8)	C1K—P1K—C7K—C8K	24.4 (8)
I2C—C3C—C4C—F2C	0.7 (12)	C19K—P1K—C7K—C12K	82.3 (8)
C2C—C3C—C4C—C5C	2.8 (14)	C13K—P1K—C7K—C12K	−38.8 (8)
I2C—C3C—C4C—C5C	−175.7 (7)	C1K—P1K—C7K—C12K	−159.0 (7)
F2C—C4C—C5C—C6C	179.9 (7)	C12K—C7K—C8K—C9K	−0.3 (14)
C3C—C4C—C5C—C6C	−3.6 (14)	P1K—C7K—C8K—C9K	176.3 (7)
F2C—C4C—C5C—I3C	1.4 (11)	C7K—C8K—C9K—C10K	−1.3 (14)
C3C—C4C—C5C—I3C	177.9 (7)	C8K—C9K—C10K—C11K	3.2 (14)
C2C—C1C—C6C—F3C	178.8 (8)	C9K—C10K—C11K—C12K	−3.5 (14)
I1C—C1C—C6C—F3C	−1.1 (11)	C8K—C7K—C12K—C11K	0.0 (14)
C2C—C1C—C6C—C5C	−2.7 (13)	P1K—C7K—C12K—C11K	−176.7 (7)
I1C—C1C—C6C—C5C	177.4 (6)	C10K—C11K—C12K—C7K	1.9 (14)
C4C—C5C—C6C—F3C	−178.0 (8)	C19K—P1K—C13K—C14K	−160.9 (7)
I3C—C5C—C6C—F3C	0.5 (11)	C7K—P1K—C13K—C14K	−38.8 (9)
C4C—C5C—C6C—C1C	3.5 (13)	C1K—P1K—C13K—C14K	79.1 (8)
I3C—C5C—C6C—C1C	−178.0 (6)	C19K—P1K—C13K—C18K	20.2 (9)
C6D—C1D—C2D—F1D	179.3 (7)	C7K—P1K—C13K—C18K	142.3 (7)
I1D—C1D—C2D—F1D	−3.3 (11)	C1K—P1K—C13K—C18K	−99.8 (8)
C6D—C1D—C2D—C3D	0.6 (13)	C18K—C13K—C14K—C15K	1.6 (14)
I1D—C1D—C2D—C3D	178.1 (7)	P1K—C13K—C14K—C15K	−177.3 (8)
F1D—C2D—C3D—C4D	−179.7 (7)	C13K—C14K—C15K—C16K	0.2 (15)
C1D—C2D—C3D—C4D	−1.0 (13)	C14K—C15K—C16K—C17K	−2.0 (15)
F1D—C2D—C3D—I2D	−4.2 (11)	C15K—C16K—C17K—C18K	2.2 (15)
C1D—C2D—C3D—I2D	174.4 (7)	C16K—C17K—C18K—C13K	−0.5 (14)
C2D—C3D—C4D—F2D	−179.3 (8)	C14K—C13K—C18K—C17K	−1.4 (14)
I2D—C3D—C4D—F2D	5.2 (12)	P1K—C13K—C18K—C17K	177.4 (7)
C2D—C3D—C4D—C5D	−0.1 (14)	C7K—P1K—C19K—C24K	163.3 (7)
I2D—C3D—C4D—C5D	−175.5 (7)	C13K—P1K—C19K—C24K	−77.1 (9)
F2D—C4D—C5D—C6D	−179.2 (8)	C1K—P1K—C19K—C24K	44.8 (9)
C3D—C4D—C5D—C6D	1.5 (14)	C7K—P1K—C19K—C20K	−18.5 (8)
F2D—C4D—C5D—I3D	−0.2 (12)	C13K—P1K—C19K—C20K	101.1 (8)
C3D—C4D—C5D—I3D	−179.4 (7)	C1K—P1K—C19K—C20K	−137.0 (7)
C4D—C5D—C6D—F3D	178.1 (8)	C24K—C19K—C20K—C21K	1.5 (14)
I3D—C5D—C6D—F3D	−0.9 (12)	P1K—C19K—C20K—C21K	−176.7 (7)
C4D—C5D—C6D—C1D	−2.0 (14)	C19K—C20K—C21K—C22K	−1.4 (15)
I3D—C5D—C6D—C1D	178.9 (7)	C20K—C21K—C22K—C23K	−0.5 (16)
C2D—C1D—C6D—F3D	−179.2 (7)	C21K—C22K—C23K—C24K	2.2 (16)
I1D—C1D—C6D—F3D	3.5 (12)	C22K—C23K—C24K—C19K	−2.0 (16)
C2D—C1D—C6D—C5D	1.0 (13)	C20K—C19K—C24K—C23K	0.1 (14)
I1D—C1D—C6D—C5D	−176.4 (7)	P1K—C19K—C24K—C23K	178.3 (8)
C6E—C1E—C2E—F1E	−179.4 (8)	C19L—P1L—C1L—C2L	91.8 (9)
I1E—C1E—C2E—F1E	−2.7 (12)	C7L—P1L—C1L—C2L	−24.5 (9)
C6E—C1E—C2E—C3E	4.6 (14)	C13L—P1L—C1L—C2L	−146.3 (8)
I1E—C1E—C2E—C3E	−178.7 (7)	C19L—P1L—C1L—C6L	−87.3 (8)
F1E—C2E—C3E—C4E	177.7 (8)	C7L—P1L—C1L—C6L	156.5 (7)
C1E—C2E—C3E—C4E	−6.3 (14)	C13L—P1L—C1L—C6L	34.7 (8)
F1E—C2E—C3E—I2E	−2.2 (11)	C6L—C1L—C2L—C3L	−1.4 (14)
C1E—C2E—C3E—I2E	173.8 (7)	P1L—C1L—C2L—C3L	179.6 (7)
C2E—C3E—C4E—F2E	−177.1 (8)	C1L—C2L—C3L—C4L	0.8 (15)
I2E—C3E—C4E—F2E	2.7 (12)	C2L—C3L—C4L—C5L	0.0 (15)
C2E—C3E—C4E—C5E	3.6 (14)	C3L—C4L—C5L—C6L	−0.4 (15)
I2E—C3E—C4E—C5E	−176.5 (7)	C4L—C5L—C6L—C1L	−0.2 (14)
F2E—C4E—C5E—C6E	−178.8 (8)	C2L—C1L—C6L—C5L	1.0 (14)
C3E—C4E—C5E—C6E	0.5 (14)	P1L—C1L—C6L—C5L	−179.9 (7)
F2E—C4E—C5E—I3E	0.0 (11)	C19L—P1L—C7L—C8L	−45.8 (8)
C3E—C4E—C5E—I3E	179.3 (7)	C13L—P1L—C7L—C8L	−168.0 (7)
C4E—C5E—C6E—F3E	178.4 (8)	C1L—P1L—C7L—C8L	73.3 (8)
I3E—C5E—C6E—F3E	−0.4 (11)	C19L—P1L—C7L—C12L	137.3 (8)
C4E—C5E—C6E—C1E	−2.4 (13)	C13L—P1L—C7L—C12L	15.2 (9)
I3E—C5E—C6E—C1E	178.8 (7)	C1L—P1L—C7L—C12L	−103.6 (8)
C2E—C1E—C6E—F3E	179.3 (8)	C12L—C7L—C8L—C9L	3.0 (14)
I1E—C1E—C6E—F3E	2.6 (11)	P1L—C7L—C8L—C9L	−173.9 (8)
C2E—C1E—C6E—C5E	0.0 (13)	C7L—C8L—C9L—C10L	−1.8 (16)
I1E—C1E—C6E—C5E	−176.7 (7)	C8L—C9L—C10L—C11L	−2.0 (16)
C6F—C1F—C2F—F1F	178.7 (8)	C9L—C10L—C11L—C12L	4.6 (16)
I1F—C1F—C2F—F1F	−3.3 (11)	C10L—C11L—C12L—C7L	−3.3 (15)
C6F—C1F—C2F—C3F	−1.5 (13)	C8L—C7L—C12L—C11L	−0.5 (14)
I1F—C1F—C2F—C3F	176.5 (6)	P1L—C7L—C12L—C11L	176.3 (7)
F1F—C2F—C3F—C4F	−177.3 (8)	C19L—P1L—C13L—C18L	−4.3 (9)
C1F—C2F—C3F—C4F	2.9 (13)	C7L—P1L—C13L—C18L	114.8 (8)
F1F—C2F—C3F—I2F	3.8 (11)	C1L—P1L—C13L—C18L	−125.8 (8)
C1F—C2F—C3F—I2F	−176.0 (6)	C19L—P1L—C13L—C14L	174.2 (7)
C2F—C3F—C4F—F2F	179.5 (8)	C7L—P1L—C13L—C14L	−66.8 (8)
I2F—C3F—C4F—F2F	−1.5 (11)	C1L—P1L—C13L—C14L	52.7 (9)
C2F—C3F—C4F—C5F	−2.5 (13)	C18L—C13L—C14L—C15L	0.1 (15)
I2F—C3F—C4F—C5F	176.5 (7)	P1L—C13L—C14L—C15L	−178.3 (8)
F2F—C4F—C5F—C6F	178.5 (8)	C13L—C14L—C15L—C16L	−0.6 (17)
C3F—C4F—C5F—C6F	0.5 (14)	C14L—C15L—C16L—C17L	1.4 (18)
F2F—C4F—C5F—I3F	1.2 (11)	C15L—C16L—C17L—C18L	−1.6 (19)
C3F—C4F—C5F—I3F	−176.8 (7)	C14L—C13L—C18L—C17L	−0.4 (14)
C2F—C1F—C6F—F3F	−179.2 (8)	P1L—C13L—C18L—C17L	178.1 (8)
I1F—C1F—C6F—F3F	2.8 (11)	C16L—C17L—C18L—C13L	1.1 (16)
C2F—C1F—C6F—C5F	−0.6 (13)	C7L—P1L—C19L—C24L	−53.0 (9)
I1F—C1F—C6F—C5F	−178.7 (7)	C13L—P1L—C19L—C24L	69.7 (9)
C4F—C5F—C6F—F3F	179.6 (8)	C1L—P1L—C19L—C24L	−170.7 (8)
I3F—C5F—C6F—F3F	−3.1 (12)	C7L—P1L—C19L—C20L	124.2 (8)
C4F—C5F—C6F—C1F	1.0 (13)	C13L—P1L—C19L—C20L	−113.2 (8)
I3F—C5F—C6F—C1F	178.3 (7)	C1L—P1L—C19L—C20L	6.4 (9)
C6G—C1G—C2G—F1G	178.6 (7)	C24L—C19L—C20L—C21L	1.6 (14)
I1G—C1G—C2G—F1G	0.1 (12)	P1L—C19L—C20L—C21L	−175.6 (8)
C6G—C1G—C2G—C3G	−1.7 (13)	C19L—C20L—C21L—C22L	−2.8 (15)
I1G—C1G—C2G—C3G	179.9 (6)	C20L—C21L—C22L—C23L	1.9 (16)
F1G—C2G—C3G—C4G	−178.6 (7)	C21L—C22L—C23L—C24L	0.2 (15)
C1G—C2G—C3G—C4G	1.6 (13)	C20L—C19L—C24L—C23L	0.5 (15)
F1G—C2G—C3G—I2G	4.1 (11)	P1L—C19L—C24L—C23L	177.7 (8)
C1G—C2G—C3G—I2G	−175.7 (7)	C22L—C23L—C24L—C19L	−1.4 (15)
C2G—C3G—C4G—F2G	178.9 (7)	C19M—P1M—C1M—C6M	139.2 (8)
I2G—C3G—C4G—F2G	−3.8 (11)	C13M—P1M—C1M—C6M	−100.9 (8)
C2G—C3G—C4G—C5G	−1.7 (13)	C7M—P1M—C1M—C6M	19.6 (9)
I2G—C3G—C4G—C5G	175.6 (6)	C19M—P1M—C1M—C2M	−46.9 (8)
F2G—C4G—C5G—C6G	−178.7 (7)	C13M—P1M—C1M—C2M	73.0 (9)
C3G—C4G—C5G—C6G	1.9 (12)	C7M—P1M—C1M—C2M	−166.5 (7)
F2G—C4G—C5G—I3G	−3.8 (11)	C6M—C1M—C2M—C3M	−1.6 (14)
C3G—C4G—C5G—I3G	176.8 (6)	P1M—C1M—C2M—C3M	−175.6 (7)
C2G—C1G—C6G—F3G	−178.6 (7)	C1M—C2M—C3M—C4M	0.3 (14)
I1G—C1G—C6G—F3G	−0.1 (10)	C2M—C3M—C4M—C5M	−0.3 (14)
C2G—C1G—C6G—C5G	1.9 (13)	C3M—C4M—C5M—C6M	1.5 (15)
I1G—C1G—C6G—C5G	−179.6 (6)	C4M—C5M—C6M—C1M	−2.8 (15)
C4G—C5G—C6G—F3G	178.5 (7)	C2M—C1M—C6M—C5M	2.9 (14)
I3G—C5G—C6G—F3G	3.4 (10)	P1M—C1M—C6M—C5M	176.5 (7)
C4G—C5G—C6G—C1G	−2.1 (12)	C19M—P1M—C7M—C12M	−13.0 (10)
I3G—C5G—C6G—C1G	−177.1 (6)	C13M—P1M—C7M—C12M	−133.7 (9)
C6H—C1H—C2H—F1H	−178.2 (8)	C1M—P1M—C7M—C12M	105.0 (9)
I1H—C1H—C2H—F1H	0.4 (11)	C19M—P1M—C7M—C8M	167.1 (8)
C6H—C1H—C2H—C3H	1.6 (13)	C13M—P1M—C7M—C8M	46.4 (10)
I1H—C1H—C2H—C3H	−179.8 (7)	C1M—P1M—C7M—C8M	−74.9 (9)
F1H—C2H—C3H—C4H	180.0 (8)	C12M—C7M—C8M—C9M	0.2 (17)
C1H—C2H—C3H—C4H	0.2 (13)	P1M—C7M—C8M—C9M	−179.9 (9)
F1H—C2H—C3H—I2H	3.7 (11)	C7M—C8M—C9M—C10M	−0.9 (19)
C1H—C2H—C3H—I2H	−176.1 (7)	C8M—C9M—C10M—C11M	2 (2)
C2H—C3H—C4H—F2H	−179.3 (8)	C9M—C10M—C11M—C12M	−3 (2)
I2H—C3H—C4H—F2H	−3.2 (12)	C8M—C7M—C12M—C11M	−0.7 (16)
C2H—C3H—C4H—C5H	−1.7 (14)	P1M—C7M—C12M—C11M	179.3 (9)
I2H—C3H—C4H—C5H	174.4 (7)	C10M—C11M—C12M—C7M	2.0 (18)
F2H—C4H—C5H—C6H	178.9 (8)	C19M—P1M—C13M—C14M	100.6 (9)
C3H—C4H—C5H—C6H	1.3 (14)	C7M—P1M—C13M—C14M	−139.4 (8)
F2H—C4H—C5H—I3H	−2.0 (12)	C1M—P1M—C13M—C14M	−17.1 (10)
C3H—C4H—C5H—I3H	−179.6 (7)	C19M—P1M—C13M—C18M	−75.4 (9)
C4H—C5H—C6H—F3H	−179.3 (8)	C7M—P1M—C13M—C18M	44.6 (10)
I3H—C5H—C6H—F3H	1.7 (13)	C1M—P1M—C13M—C18M	166.9 (8)
C4H—C5H—C6H—C1H	0.7 (15)	C18M—C13M—C14M—C15M	−0.3 (15)
I3H—C5H—C6H—C1H	−178.3 (7)	P1M—C13M—C14M—C15M	−176.2 (7)
C2H—C1H—C6H—F3H	177.9 (8)	C13M—C14M—C15M—C16M	−0.7 (14)
I1H—C1H—C6H—F3H	−0.6 (12)	C14M—C15M—C16M—C17M	2.3 (15)
C2H—C1H—C6H—C5H	−2.1 (14)	C15M—C16M—C17M—C18M	−3.0 (17)
I1H—C1H—C6H—C5H	179.4 (7)	C16M—C17M—C18M—C13M	2.0 (17)
C13I—P1I—C1I—C2I	132.8 (8)	C14M—C13M—C18M—C17M	−0.4 (17)
C19I—P1I—C1I—C2I	−106.5 (8)	P1M—C13M—C18M—C17M	175.7 (9)
C7I—P1I—C1I—C2I	11.3 (9)	C13M—P1M—C19M—C24M	−162.7 (7)
C13I—P1I—C1I—C6I	−48.6 (8)	C7M—P1M—C19M—C24M	77.8 (8)
C19I—P1I—C1I—C6I	72.1 (8)	C1M—P1M—C19M—C24M	−43.2 (9)
C7I—P1I—C1I—C6I	−170.2 (7)	C13M—P1M—C19M—C20M	20.3 (9)
C6I—C1I—C2I—C3I	0.8 (15)	C7M—P1M—C19M—C20M	−99.2 (8)
P1I—C1I—C2I—C3I	179.3 (8)	C1M—P1M—C19M—C20M	139.8 (8)
C1I—C2I—C3I—C4I	−1.4 (16)	C24M—C19M—C20M—C21M	1.3 (14)
C2I—C3I—C4I—C5I	0.4 (16)	P1M—C19M—C20M—C21M	178.4 (8)
C3I—C4I—C5I—C6I	1.2 (16)	C19M—C20M—C21M—C22M	−0.8 (16)
C4I—C5I—C6I—C1I	−1.8 (15)	C20M—C21M—C22M—C23M	−0.2 (16)
C2I—C1I—C6I—C5I	0.8 (14)	C21M—C22M—C23M—C24M	0.8 (15)
P1I—C1I—C6I—C5I	−177.8 (7)	C20M—C19M—C24M—C23M	−0.8 (14)
C13I—P1I—C7I—C12I	−10.8 (9)	P1M—C19M—C24M—C23M	−177.9 (8)
C1I—P1I—C7I—C12I	108.6 (8)	C22M—C23M—C24M—C19M	−0.2 (15)
C19I—P1I—C7I—C12I	−131.7 (7)	C19N—P1N—C1N—C6N	−24.0 (10)
C13I—P1I—C7I—C8I	171.8 (7)	C7N—P1N—C1N—C6N	96.7 (9)
C1I—P1I—C7I—C8I	−68.8 (8)	C13N—P1N—C1N—C6N	−144.7 (8)
C19I—P1I—C7I—C8I	50.9 (8)	C19N—P1N—C1N—C2N	160.7 (8)
C12I—C7I—C8I—C9I	−2.1 (14)	C7N—P1N—C1N—C2N	−78.6 (9)
P1I—C7I—C8I—C9I	175.4 (8)	C13N—P1N—C1N—C2N	40.0 (9)
C7I—C8I—C9I—C10I	0.0 (15)	C6N—C1N—C2N—C3N	−0.9 (15)
C8I—C9I—C10I—C11I	2.0 (16)	P1N—C1N—C2N—C3N	174.6 (8)
C9I—C10I—C11I—C12I	−2.0 (16)	C1N—C2N—C3N—C4N	1.3 (16)
C8I—C7I—C12I—C11I	2.1 (13)	C2N—C3N—C4N—C5N	−0.9 (16)
P1I—C7I—C12I—C11I	−175.2 (7)	C3N—C4N—C5N—C6N	0.0 (15)
C10I—C11I—C12I—C7I	0.0 (15)	C4N—C5N—C6N—C1N	0.4 (15)
C1I—P1I—C13I—C14I	−55.0 (8)	C2N—C1N—C6N—C5N	0.1 (15)
C19I—P1I—C13I—C14I	−175.6 (7)	P1N—C1N—C6N—C5N	−175.3 (8)
C7I—P1I—C13I—C14I	65.5 (8)	C19N—P1N—C7N—C8N	−45.6 (10)
C1I—P1I—C13I—C18I	121.5 (8)	C1N—P1N—C7N—C8N	−167.3 (9)
C19I—P1I—C13I—C18I	1.0 (9)	C13N—P1N—C7N—C8N	74.2 (10)
C7I—P1I—C13I—C18I	−118.0 (8)	C19N—P1N—C7N—C12N	135.2 (8)
C18I—C13I—C14I—C15I	1.6 (14)	C1N—P1N—C7N—C12N	13.4 (9)
P1I—C13I—C14I—C15I	178.3 (8)	C13N—P1N—C7N—C12N	−105.0 (8)
C13I—C14I—C15I—C16I	−2.6 (16)	C12N—C7N—C8N—C9N	3.2 (17)
C14I—C15I—C16I—C17I	1 (2)	P1N—C7N—C8N—C9N	−176.1 (9)
C15I—C16I—C17I—C18I	2 (2)	C7N—C8N—C9N—C10N	−3.0 (19)
C16I—C17I—C18I—C13I	−2.8 (19)	C8N—C9N—C10N—C11N	1.3 (17)
C14I—C13I—C18I—C17I	1.1 (16)	C9N—C10N—C11N—C12N	0.3 (16)
P1I—C13I—C18I—C17I	−175.5 (9)	C10N—C11N—C12N—C7N	−0.1 (15)
C13I—P1I—C19I—C20I	114.8 (7)	C8N—C7N—C12N—C11N	−1.6 (14)
C1I—P1I—C19I—C20I	−4.3 (8)	P1N—C7N—C12N—C11N	177.7 (7)
C7I—P1I—C19I—C20I	−123.6 (7)	C19N—P1N—C13N—C14N	89.4 (9)
C13I—P1I—C19I—C24I	−68.1 (8)	C1N—P1N—C13N—C14N	−149.4 (8)
C1I—P1I—C19I—C24I	172.8 (7)	C7N—P1N—C13N—C14N	−29.8 (10)
C7I—P1I—C19I—C24I	53.4 (8)	C19N—P1N—C13N—C18N	−87.3 (9)
C24I—C19I—C20I—C21I	1.2 (13)	C1N—P1N—C13N—C18N	33.9 (10)
P1I—C19I—C20I—C21I	178.2 (6)	C7N—P1N—C13N—C18N	153.5 (8)
C19I—C20I—C21I—C22I	1.2 (13)	C18N—C13N—C14N—C15N	1.1 (15)
C20I—C21I—C22I—C23I	−2.8 (14)	P1N—C13N—C14N—C15N	−175.6 (8)
C21I—C22I—C23I—C24I	1.9 (15)	C13N—C14N—C15N—C16N	0.7 (17)
C22I—C23I—C24I—C19I	0.6 (16)	C14N—C15N—C16N—C17N	−3.4 (18)
C20I—C19I—C24I—C23I	−2.1 (14)	C15N—C16N—C17N—C18N	4.2 (18)
P1I—C19I—C24I—C23I	−179.2 (8)	C16N—C17N—C18N—C13N	−2.2 (17)
C13J—P1J—C1J—C6J	112.5 (7)	C14N—C13N—C18N—C17N	−0.4 (16)
C19J—P1J—C1J—C6J	−127.3 (7)	P1N—C13N—C18N—C17N	176.4 (8)
C7J—P1J—C1J—C6J	−7.5 (8)	C1N—P1N—C19N—C20N	82.1 (10)
C13J—P1J—C1J—C2J	−68.2 (8)	C7N—P1N—C19N—C20N	−39.2 (11)
C19J—P1J—C1J—C2J	51.9 (8)	C13N—P1N—C19N—C20N	−158.2 (9)
C7J—P1J—C1J—C2J	171.7 (7)	C1N—P1N—C19N—C24N	−95.9 (9)
C6J—C1J—C2J—C3J	−4.4 (14)	C7N—P1N—C19N—C24N	142.8 (8)
P1J—C1J—C2J—C3J	176.4 (7)	C13N—P1N—C19N—C24N	23.8 (10)
C1J—C2J—C3J—C4J	5.3 (14)	C24N—C19N—C20N—C21N	1.2 (19)
C2J—C3J—C4J—C5J	−4.0 (16)	P1N—C19N—C20N—C21N	−176.9 (10)
C3J—C4J—C5J—C6J	1.4 (16)	C19N—C20N—C21N—C22N	1 (2)
C4J—C5J—C6J—C1J	−0.3 (14)	C20N—C21N—C22N—C23N	−3 (2)
C2J—C1J—C6J—C5J	1.9 (13)	C21N—C22N—C23N—C24N	1.8 (19)
P1J—C1J—C6J—C5J	−178.9 (7)	C22N—C23N—C24N—C19N	0.8 (17)
C1J—P1J—C7J—C12J	−102.8 (8)	C20N—C19N—C24N—C23N	−2.3 (16)
C13J—P1J—C7J—C12J	135.3 (8)	P1N—C19N—C24N—C23N	175.7 (8)

### Hydrogen-bond geometry (Å, º)

**Table:**

*D*—H···*A*	*D*—H	H···*A*	*D*···*A*	*D*—H···*A*
C20*I*—H20*I*···I3^i^	0.95	3.16	3.866 (9)	133
C20*L*—H20*L*···I6	0.95	3.09	3.821 (9)	135
C14*M*—H14*M*···I3^ii^	0.95	3.14	3.788 (9)	127
C8*L*—H8*L*···F3*F*^i^	0.95	2.59	3.351 (10)	138
C20*L*—H20*L*···I6	0.95	3.09	3.821 (9)	135
C18*M*—H18*M*···F2*C*^iii^	0.95	2.56	3.257 (11)	130
C18*L*—H18*L*···O2*S*	0.95	2.61	3.25 (2)	125
O1*S*—H1*S*···I1	0.85	2.71	3.561 (15)	177
O2*S*—H2*S*···O1*S*	0.85	1.83	2.68 (3)	176

Symmetry codes: (i) *x*−1, *y*, *z*; (ii) *x*−1, *y*+1, *z*; (iii) −*x*+1, *y*+1/2, −*z*+1.

### Short C—I···I^-^ contacts and XBs (Å, °).

The table is organized to evidence the difference between the two pseudo-centrosymmetric units: similar contacts are on the same line and are also reported if they are too long to be considered XBs.

**Table:**

C—*X*···*Y*	*X*···*Y*	C—*X*···*Y*	C—*X*···*Y*	*X*···*Y*	C—*X*···*Y*
C1A—I1A···I1	3.5318 (10)	175.26 (3)	C1E—I1E···I4	3.5567 (10)	177.55 (3)
C3E—I2E···I1	3.6860 (10)	169.40 (3)	C3A—I2A···I4	3.5578 (11)	173.31 (3)
C1B—I1B···I1	3.4466 (9)	169.04 (3)	C1F—I1F···I4	3.4131 (10)	168.78 (3)
C3G—I2G···I1	3.4582 (10)	169.32 (2)	C3C—I2C···I4	3.4102 (10)	172.40 (3)
C5A—I3A···I2	3.4725 (10)	167.88 (3)	C5E—I3E···I5	3.4221 (10)	174.57 (3)
C3D—I2D···I2	3.4963 (10)	174.57 (3)	C3H—I2H···I5	3.5754 (10)	168.90 (3)
C5B^i^—I3B^i^···I2	3.5678 (10)	171.87 (3)	C5F^ii^—I3F^ii^···I5	3.5696 (10)	160.64 (3)
C5C^i^—I3C^i^···I2	3.5029 (9)	164.46 (3)	C5G^ii^—I3G^ii^···I5	3.6253 (10)	157.18 (3)
C5D^i^—I3D^i^···I2	3.6906 (10)	176.88 (3)	C5H^ii^—I3H^ii^···I5	4.0145 (11)	166.52 (3)
C1C—I1C···I3	3.2760 (9)	179.47 (3)	C1H^ii^—I1H^ii^···I3	3.3977 (10)	178.17 (3)
C1G—I1G···I6	3.2889 (9)	172.31 (3)	C1D^i^—I1D^i^···I6	3.4609 (10)	172.84 (3)

Symmetry codes: (i) 1-x, 1/2+y, 1-z; (ii) 2-x, -1/2+y, 2-z.

### HB distances and angles (Å, °).

**Table:**

D-H···A	D-H	H···A	D-H···A	D···A
C20I—H20I···I3^i^	0.950	3.159	132.69	3.866
C20L—H20L···I6	0.950	3.088	135.18	3.821
C14M—H14M···I3^ii^	0.950	3.140	126.92	3.788
C8L—H8L···F3F^i^	0.950	2.588	137.54	3.351
C20L—H20L···I6	0.950	3.088	135.18	3.821
C18M—H18M···F2C^iii^	0.950	2.560	130.41	3.257
C18L—H18L···O2S	0.950	2.612	124.50	3.246
O1S—H1S···I1	0.850	2.711	177.36	3.561
O2S—H2S···O1S	0.850	1.831	176.35	2.679

Symmetry codes: (i) -1+x, y, z; (ii) x, -1+y, z; (iii) -1+x, 1+y, 1-z.
